# Potential network markers and signaling pathways for B cells of COVID-19 based on single-cell condition-specific networks

**DOI:** 10.1186/s12864-023-09719-1

**Published:** 2023-10-18

**Authors:** Ying Li, Liqin Han, Peiluan Li, Jing Ge, Yun Xue, Luonan Chen

**Affiliations:** 1 School of Mathematics and Statistics, Henan University of Science and Technology, Luoyang, 471023 China; 2 Longmen Laboratory, Luoyang, 471003 Henan China; 3 Shanghai Immune Therapy Institute, Renji Hospital, Shanghai Jiao Tong University School of Medicine, Shanghai, 200032 China; 4 College of Medical Technology and Engineering, Henan University of Science and Technology, Luoyang, 471023 China; 5 Key Laboratory of Systems Biology, Institute of Biochemistry and Cell Biology, Center for Excellence in Molecular Cell Science, Chinese Academy of Sciences, Shanghai, 201100 China; 6 Key Laboratory of Systems Health Science of Zhejiang Province, Hangzhou Institute for Advanced Study, University of Chinese Academy of Sciences, Hangzhou, 310000 China; 7 School of Life Science and Technology, ShanghaiTech University, Shanghai, 201100 China; 8 West China Biomedical Big Data Center, Med-X Center for Informatics, West China Hospital, Sichuan University, Chengdu, 610041 China

**Keywords:** Single-cell RNA sequencing, B cells, Conditional cell-specific network, Hub gene, ‘Dark’ genes, Pathway

## Abstract

To explore the potential network markers and related signaling pathways of human B cells infected by COVID-19, we performed standardized integration and analysis of single-cell sequencing data to construct conditional cell-specific networks (CCSN) for each cell. Then the peripheral blood cells were clustered and annotated based on the conditional network degree matrix (CNDM) and gene expression matrix (GEM), respectively, and B cells were selected for further analysis. Besides, based on the CNDM of B cells, the hub genes and ‘dark’ genes (a gene has a significant difference between case and control samples not in a gene expression level but in a conditional network degree level) closely related to COVID-19 were revealed. Interestingly, some of the ‘dark’ genes and differential degree genes (DDGs) encoded key proteins in the JAK-STAT pathway, which had antiviral effects. The protein p21 encoded by the ‘dark’ gene CDKN1A was a key regulator for the COVID-19 infection-related signaling pathway. Elevated levels of proteins encoded by some DDGs were directly related to disease severity of patients with COVID-19. In short, the proteins encoded by ‘dark’ genes complement some missing links in COVID-19 and these signaling pathways played an important role in the growth and activation of B cells.

**Supplementary Information:**

The online version contains supplementary material available at 10.1186/s12864-023-09719-1.

## Background

Acute infection with severe acute respiratory syndrome coronavirus 2 (SARS-CoV-2) has rapidly caused the ongoing worldwide pandemic of coronavirus disease 2019 (COVID-19). COVID-19 can present with a spectrum of illness, from asymptomatic, mild, moderate, to severe and death [1, 2]. Disease outcome is dictated by a combination of direct viral effects on patient tissues [3], protective antiviral immunity [4] and overexuberant antiviral or inflammatory immune responses driving tissue damage [5, 6]. However, it is unclear what causes moderate to severe symptoms in some patients. How communication between host compartments controls the disease progression. And whether enriched pathways are involved in antiviral immune responses or not.

Since the outbreak of COVID-19, the research related to SARS-CoV-2 based on single-cell sequencing has drawn more and more attention. Zhao et al. [7] found that both SARS virus infection and the S protein of SARS virus could reduce the expression of ACE2 protein and lead to acute lung failure in vivo, revealing that the lower respiratory tract is the target organ attacked by SARS-COV-2 at the single-cell level. Ren et al. [8] integrated and analyzed the single-cell data of COVID-19 and found that the presence of SARS-CoV-2 RNA in various immune cell types, including neutrophils, macrophages, plasma B cells, T cells, and NK cells. Reveal the mechanism of COVID-19 infection and the characteristics of the body's immune response in different disease stages. Cristinelli et al. [9] found that the expression of host genes at the transcriptional level under virus infection was helpful for the discovery of virus infection-related markers and the understanding of the mechanism of virus-host interaction. Zou et al. [10] found that the SARS-CoV-2 first attacked the lungs, but it was clinically found that the SARS-CoV-2 appeared in other organs, such as the heart and kidneys. Zhang et al. [11] constructed a gene co-expression network based on single-cell sequencing data of patients with COVID-19 infection, and found a group of functional genes with similar expression patterns with EEF1A1 as the center, which was expected to be used as a marker for diagnosis and treatment of COVID-19 infection. Severe COVID-19 patients have severe inflammatory response [12], innate immunity [13] and impaired adaptive immune response [14–16]. These studies deepen our understanding of the disease’s immunopathology.

The above-mentioned study mainly focused on the analyses of gene expression levels. In order to further reveal some potential pathogenic mechanisms of COVID-19, we studied COVID-19 from the direct gene–gene association levels. Dai et al. [17] presented a new method to construct a cell-specific network (CSN) for each single cell from scRNA-seq data (i.e., one network for one cell), which transformed the data from ‘unstable’ gene expression form to ‘stable’ gene association form on a single-cell basis. Based on network degree matrix (NDM), CSN could be further applied to downstream single-cell analysis, and its effectiveness in terms of robustness and accuracy is validated on multiple scRNA-seq datasets. In 2021, Li et al. [18] proposed CCSN to identify direct associations between genes by filtering out indirect associations in the gene–gene network based on conditional independence, which overcame the disadvantage that some genes that were not directly related might be wrongly judged as related in the CCSN.

In this study, we constructed a CCSN for each cell in the HRA000150 dataset, in which each gene was represented as a node in the network, and the correlation between genes was represented as an edge in the network. CCSN can transform the original gene expression data of each cell to the direct and robust gene–gene association data (or network data) of the same cell. CCSN also offers an approach for identifying pivotal genes from a network viewpoint. Given that pivotal regulatory genes typically exert influence on the expression of numerous other genes, they tend to exhibit a greater number of connections to other genes within CCSNs. This results in a higher network degree for these pivotal genes. By quantifying the number of connections (known as the network degree) for each gene within each CCSN, we can identify the genes with the highest degrees in each cell or cell type. These highest-degree genes serve as representative key genes when considering the network perspective. We converted CCSN to CNDM by calculating the network degree of each gene in the cell. CNDM can reduce dimensions while integrating high-dimensional single-cell network data. Moreover, the CNDM matrix could be further analyzed by most traditional scRNA-seq methods for dimension reduction and clustering analysis. Then B cells were extracted for subsequent analysis. CNDM serves as a measure of the significance of each gene within the network and shares the same dimension as the gene expression matrix (GEM). The CCSN method can reduce noise and improve coverage. Each CCSN can be viewed as the transformation from less ‘reliable’ gene expression to more ‘reliable’ gene–gene associations in a cell. In addition, CCSNs found hub genes in different stages of COVID-19 from a network viewpoint, even ‘dark’ genes, which had no significantly differential changes in terms of gene expression, and thus couldn’t be found by traditional differential analyses. But they had significant differential changes in terms of network degree, therefore may also play an important role in the network regulation. We validated ‘dark’ genes at protein level and performed prognostic analysis of ‘dark’ genes, which showed that ‘dark’ genes play an important role in the progress of COVID-19. ‘Dark’ genes were enriched in some pathways, and some proteins encoded by ‘dark’ genes were involved in a series of signaling pathway, which revealed the relevant roles of ‘dark’ genes in SARS-CoV-2 infection and COVID-19 treatment. Our findings might be helpful for understanding and controlling COVID-19.

## Methods

### Data pre-processing

We downloaded the raw single-cell peripheral blood sequencing data from the Genome Sequence Archive of the Beijing Institute of Genomics (BIG) Data Center (http://bigd.big.ac.cn/gsa-human, accession number: HRA000150). The raw scRNA-seq FASTQ files of PBMCs from 13 patients and 5 healthy controls. The 13 patients with COVID-19 were classified into three clinical conditions: moderate (*n* = 7), severe (*n* = 4) and convalescent (conv; *n* = 6, of whom 4 were paired with moderate cases). The downloaded reads were then processed individually by using the Cell Ranger (v.4.0.0, 10xgenomics, https://www.10xgenomics.com/) to count pipeline with the GRCh38 human reference genome to generate GEM. The subsequent analyses were performed by R (v.4.1.0) scripts with the Seurat (v.3.2.2) package. Briefly, GEMs filtered by the Cell Ranger were further filtered based on three metrics step by step: the total UMI counts, number of detected genes and proportion of mitochondrial gene counts per cell [19]. We separately integrated healthy, moderate, severe, and recovery stage samples to obtain the initial GEMs. This study also included 171 patients with COVID-19 and 25 healthy individuals from 37 centers / laboratories, with samples (*n* = 284) collected (http://bigd.big.ac.cn/gsa-human, accession number: HRA001149). Samples of COVID-19 were further categorized into groups of moderate convalescence (*n* = 89), moderate progression (*n* = 33), severe convalescence (*n* = 51) and severe progression (*n* = 83). And CITE-seq of 7 COVID-19 patients and 5 healthy controls (https://www.ncbi.nlm.nih.gov/geo/, accession number: GSE155673), of which 7 COVID-19 patients were 4 severe stage and 3 mild stage. The logarithm $\log (1 + x)$ was applied to normalize the initial GEMs with $M$ rows/genes and $N$ columns/cells [20, 21].

### Construction of CCSN

From dynamical viewpoint, the network of a cell could more reliably characterize the biological system or state of the cell. Li et al. proposed to construct CCSNs for each cell based on probability theory [18], which could measure the direct associations between genes by eliminating the indirect associations. Each CCSN could be viewed as the transformation from less ‘reliable’ gene expression to more ‘reliable’ gene–gene associations in a cell and the problem of dropout events in scRNA-seq data were somewhat alleviated by increasing gene–gene associations.

**Fig. 1:**
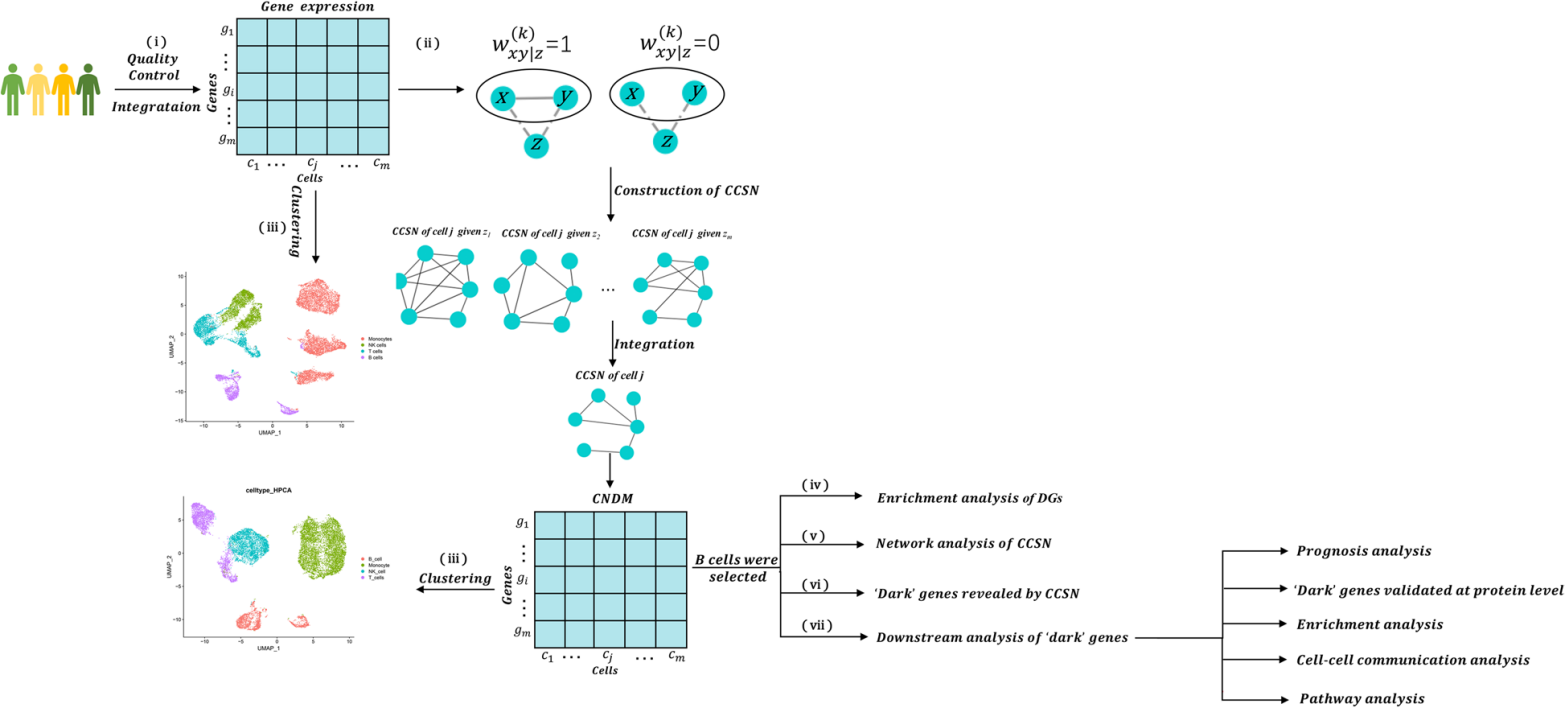
The schematic illustration of the research analysis. (i)The samples of the four stages were integrated, and a series of quality controls were carried out to obtain the GEMs. (ii)Construct CCSNs based on gene expression data at different stages. (iii) Display of cell clustering results of GEM and CCSN at different stages. (iv) B cells were selected for further analysis. Based on CCSN and GEM, common genes with significant differences between different stages of COVID-19 were found and downstream analyses were performed. (v) Demonstrate network dynamics at different stages. (vi) Find ‘dark’ genes with no significant differences at the gene expression level but significant differences at the network degree level. (vii) Perform downstream analysis of ‘dark’ genes

Due to the large number of cell samples in the single-cell sequencing data, the dataset obtained after preprocessing the original data contained four stages, including 12,537 cells in the healthy, 16,297 cells in the moderate, 14,900 cells in the severe and 10,346 cells in convalescent. For the data preprocessing, we first calculated the average expression level of each gene, and took the top 5000 genes for subsequent analysis to ease the calculation pressure and reduce the low expression level. CCSNs were constructed for 5000 different stages of gene expression profiling data. A general overview of the study is visualized in the following flow diagram to illustrate our analysis process is shown in Fig. 1.

### Enrichment analysis of DGs

To explore the differential genes of B cells under SARS-COV-2 infection, dimension reduction and clustering analysis were performed on CNDM derived from CCSN, and B cells were extracted for subsequent analysis. The healthy controls were selected for comparative analysis with moderate, severe and convalescent, respectively, and DDGs of three stages were obtained. The common differential genes of these three stages were used as DDGs of B cells, and subsequent analysis was based on DDGs. The screening criteria for DDGs were adjusted *p*-value < 0.05 and average fold change |avg_log2FC|> 0.25. Similarly, the differential expression genes (DEGs) of B cells in GEM were also found, and the common genes of DDGs and DEGs were collectively referred to as differential genes (DGs). To determine the biological functions of DGs, we used Gene Ontology (GO) and Kyoto Encyclopedia of Genes and Genomes (KEGG) pathway enrichment analysis to find the important biological functions of the DGs and the processes related to the development of COVID-19 that the DGs participated in. To enhance the robustness of the results, we integrated three datasets, HRA000150, HRA001149 and GSE155673, into the analysis. We used the integrated data for differential expression analysis (logfc.threshold = 0.15) to obtain DEGs and then performed enrichment analysis.

### Network analysis of CCSN

CCSN provides a new way to build gene–gene interaction networks in each cell and find hub genes from a network perspective. By counting the number of edges connected by each gene (i.e., the conditional network degree) in each CCSN, we selected the gene with the highest degree in B cells of severe stage as the hub gene from the network perspective. And the CNDM derived from CCSNs could be further used in dimension reduction and clustering analysis by many existing methods. We use PCA [22] and t-SNE [23], which represent linear and nonlinear methods, respectively, to perform dimension reduction on public scRNA-seq datasets with known cell types. We used Seurat package to cluster scRNA-seq data and visualize the clustering results with UMAP.

### ‘Dark’ genes revealed by CNDM

In the field of biomedicine, DEGs play an important role in finding new biomarkers, key regulators and drug targets, and some non-DEGs may also be involved in important biological processes and should not be ignored. If a gene has a significant difference between case and control samples not in a gene expression level but in a conditional network degree level, we called this gene as ‘dark’ gene. By CNDM, we were able to reveal the ‘dark’ genes, which were of great importance in the network regulation, and they could not be found by traditional differential analyses [18].

### Downstream analysis of ‘dark’ genes

Downstream analysis of ‘dark’ genes mainly included prognostic analysis, protein level validation, enrichment analysis, cell–cell communication analysis and pathway analysis. Through the validation at protein level and prognostic analysis, the biological functions of ‘dark’ genes were proved to be related to COVID-19. Cell–cell communication analysis the B cells were performed. We found that some ‘dark’ genes were present in the most interacting receptors, and these ‘dark’ genes were further clustered by expression level. And the ‘dark’ genes were further validated by the enrichment analysis, and pathway analysis, which showed that ‘dark’ genes were involved in biological processes associated with COVID-19.

## Results

### Construct of CCSN and obtain CNDM from CCSN

To overcome the problem of overestimation of the related indirect effects between genes in each cell, Li et al. proposed the c-CSN method, which could construct a CCSN for each cell [18]. The CCSN could identify direct associations between a pair of genes in a cell by eliminating indirect associations between genes by selecting a small number of conditional genes.

The statistical dependency index $\rho_{xy|z}^k$ defined in Supplementary Note 1. If $\rho_{xy|z}^k$ is greater than zero, there is an edge between $x$ and $y$ in the cell $k$, otherwise, there is no edge. In this way, we construct a CCSN for each cell, where edge between two gene $x$ and $y$ is decided by the dependency index $\rho_{xy|z}^k$. Then we obtain gene–gene interaction network. Through the corresponding formula transformation, we get CNDM. The matrix has the same dimension with the GEM. CNDM can reflect the gene–gene direct association in terms of interaction degrees. Moreover, after normalization, this CNDM matrix could be further analyzed by most traditional scRNA-seq methods for dimension reduction and clustering analysis. The input and output settings of CCSN method are listed in Supplementary Note 1. The detailed description of algorithm for constructing CCSN is provided in Supplementary Note 1.

### Distribution of B cells in peripheral blood cells and cell counts

The CCSN reflected not the level of gene expression, but the degree of association of each gene in the network, and the CNDM had the same dimension with the GEM. When performing cell type clustering analysis, we only needed to replace the original GEM with CNDM. Then we could use any traditional scRNA-seq algorithm for cell clustering and dimensionality reduction, which opened up a new way for us to analyze scRNA-seq data from the network level.

**Fig. 2:**
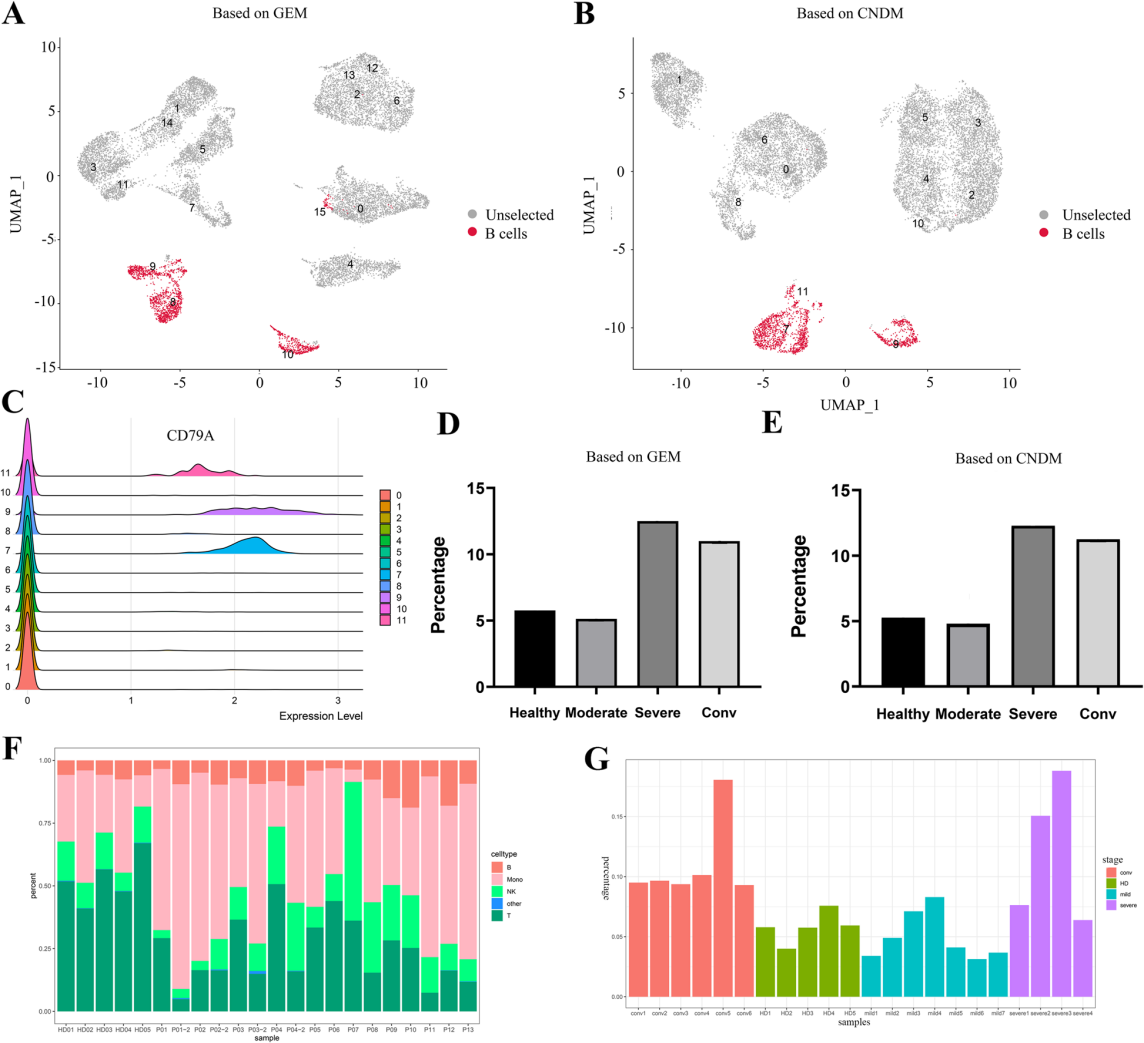
Distribution of B cells in peripheral blood cells and cell counts. **A** UMAP projection of the GEM of the dataset HRA000150. UMAP projection representing B cells in pink color and the rest of the cells in grey color. **B** UMAP projection of the CNDM of the dataset HRA000150. **C** Ridge diagram annotates B cells according to the conditional network degree levels of the canonical marker CD79A. **D** Bar plot representing the percentage of B cells among all cell types of each condition based on GEM. **E** Bar plot representing the percentage of B cells among all cell types of each condition based on CNDM. **F** Bar graphs showing the percentage of monocyte, NK cells, T cells, and B cells in each sample. **G** Bar plot representing the percentage of B cells among all cell types of each sample based on GEM

The B cells of the immune system in COVID-19 patients were fully fighting the SARS-CoV-2 virus. The key characteristics of B cells were effective at neutralizing, or inactivating, the SARS-CoV-2 virus and related coronaviruses [24]. In order to better study B cells, we performed cell clustering and dimensionality reduction based on GEM and CNDM, respectively (Supplementary Fig. 1A), and annotated B cells according to the conditional network degree levels of the canonical marker CD79A (Fig. 2C and Supplementary Fig. 1B). Other cell types were also annotated based on the conditional network degree levels of canonical markers (Supplementary Fig. 1B, C). Figure 2A and B show the distribution of B cells in the whole peripheral blood cells of severe COVID-19 patients. Meanwhile, it can be seen from the figure that the B cell distribution obtained from cell clustering based on CNDM is more concentrated, probably because the CCSN method performs better than the conventional methods (Fig. 2A, B). We performed B cell counts based on GEM, and the percentage of cells increased from 5.77% in healthy individuals to 12.50% in severe cases as shown in Fig. 2D. We performed B cell counts based on CNDM, and the percentage of B cells increased from 5.26% in healthy individuals to 12.28% in severe cases as shown in Fig. 2E. So the percentage of B cells among all population in severe COVID-19 cases was found to be significantly enhanced (Fig. 2D, E). Besides, we also performed cell counts on monocytes, NK cells and T cells (Supplementary Fig. 1D). The results showed that the percentage of B cells changed more significantly compared to the percentages of other cell types. Simultaneously, the percentage of B cells was also reported to be high in COVID-19 patients and increased with disease severity [25]. To ensure that the findings were not driven by outlier cells within a single sample, monocytes, NK, T and B cells were counted at the sample level by GEM (Fig. 2F*)*. Meanwhile, we counted B cells at the sample level, and B cell counts changed slightly from healthy to moderate stage, rose sharply to severe stage, and finally decreased and maintained a stable during the convalescence stage. This conclusion is in consistent with what we found at the individual cell level (Fig. 2G). Therefore, we selected B cells for subsequent analysis.

### GO enrichment analysis of differential genes

**Fig. 3:**
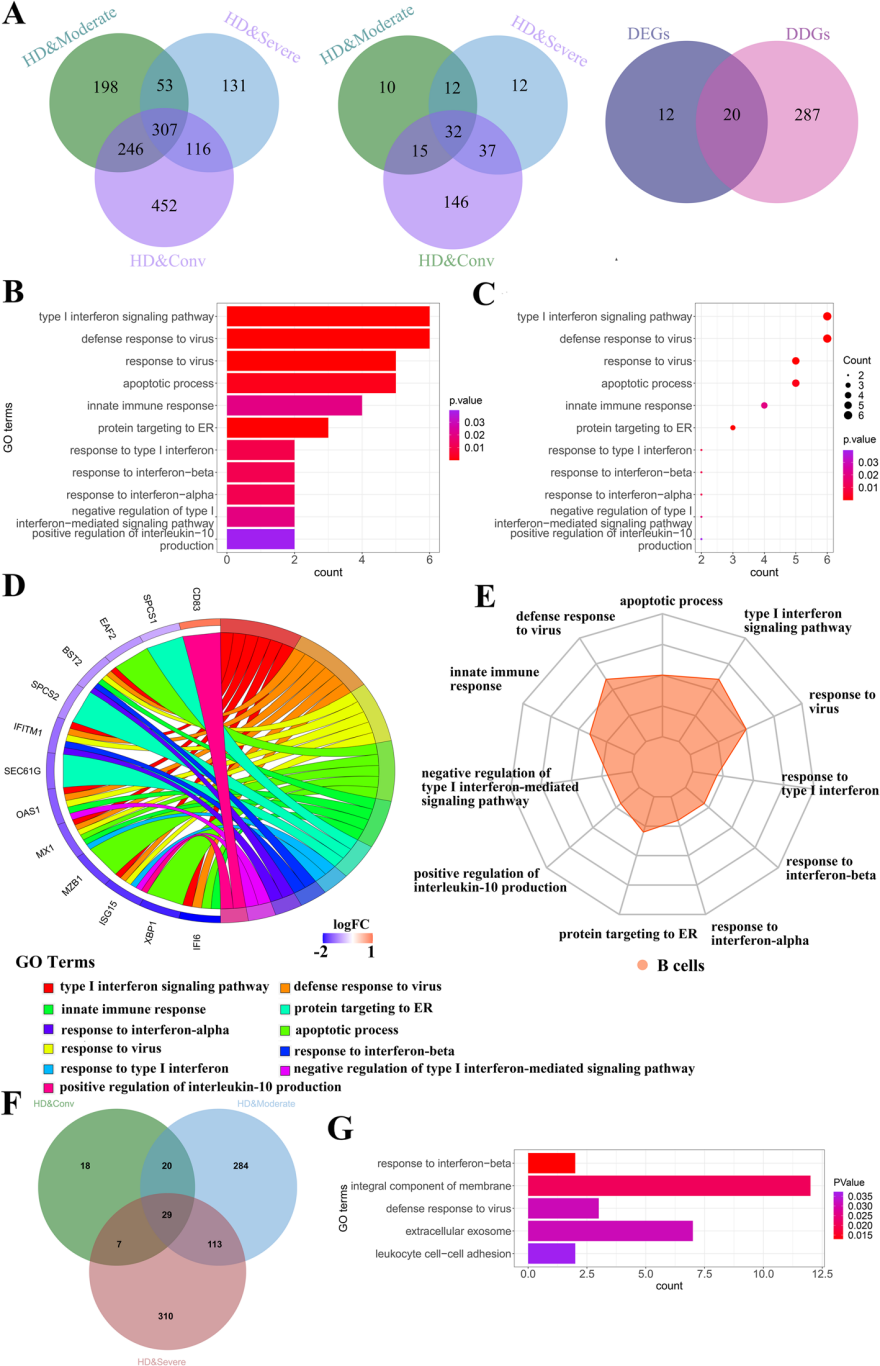
Gene enrichment analyses of the DGs.** A** Venn diagram showing the overlapping DDGs among differential degree genes in B cells of four different stages based on CNDM, the overlapping DEGs among differential expression genes in B cells of four different stages based on GEM, and the overlapping DGs among DDGs and DEGs. **B** Bar plot of enriched GO terms for DGs. GO terms are labeled with name and ID and are sorted by the number of genes enriched in the GO terms. the color indicates the *p*-value. **C** Dot plot of enriched GO terms. **D** The GO chord plot shows the 13 DGs and the 11 enriched GO terms.** E** Radar chart of enriched GO terms. **F** Venn diagram showing the overlap between differentially expressed genes in B cells at four different stages based on the integrated GEM. **G** Bar plot of enriched GO terms for integrated DEGs. GO terms are labeled with name and ID and are sorted by the number of genes enriched in the GO terms. the color indicates the *p*-value

Through the FindMarkers function, 307 common DDGs were found in B cells of four different stages based on CNDM, and 32 common DEGs were found in B cells of four different stages based on GEM. Next, we obtained 20 DGs, which are the common genes of DDGs and DEGs (Fig. 3A, Supplementary Table S1). Then we performed GO and KEGG enrichment analysis on DGs (Supplementary Table S2). Because of the significant differences in both gene expression level and conditional network degree level, these 20 DGs might have important biological significance in COVID-19. As shown in Table 1, some DGs were enriched into the biological processes of immunity or defense against the SARS-COV-2, e.g. ‘response to type I interferon’ (GO:0034340), ‘defense response to virus’ (GO:0051607), ‘innate immune response’ (GO:0045087), ‘positive regulation of interleukin-10 production’ (GO:0032733), ‘apoptotic process’ (GO:0006915), ‘response to interferon-alpha’ (GO:0035455), ‘response to interferon-beta’ (GO:0035456) and others in Gene ontology (Fig. 3B-E). Meanwhile, as shown in Table 2, these DGs are involved in some KEGG pathway, e.g. ‘protein export’ (hsă0) and ‘Coronavirus disease—COVID-19’ (hsa05171). Type I interferons (IFN-I), mainly represented by IFN-α and β, are a group of cytokines with an important function in antiviral responses and have played a complex role in COVID-19. The antiviral effects of interferons include inhibition of viral replication, protein synthesis, maturation, and release. Some studies showed that in moderate cases, IFN-I levels and interferon responses were elevated. While other studies noted that in severe cases, IFN-I levels and interferon responses were elevated. In the study of the severity of COVID-19, some researchers summarized the correlation between plasma type I interferon levels and the severity of COVID-19 [26]. To ensure that the results do not only rely on a single study with a limited sample size, further improving the robustness and generalizability of the results, we integrated the three datasets. Then the differential expression analysis of the integrated gene expression data yielded 29 DEGs (Fig. 3F and Supplementary Table S6). Next, we performed an enrichment analysis on these differential genes. As shown in Fig. 3G. Some DEGs were enriched into the biological processes of immunity or defense against the SARS-COV-2, e.g. ‘response to interferon-beta’(GO:0035456), ‘integral component of membrane’(GO:0016021), ‘defense response to virus’(GO:0051607),‘extracellular exosome’(GO:0070062), ‘leukocyte cell–cell adhesion’ (GO:0007159). Similar to other coronaviruses, SARS-CoV-2 utilized the host cell’s secretory apparatus to generate viral proteins, which constituted the emerging viral particles. These proteins, namely spike (S), envelope (E), and membrane (M) proteins, were the transmembrane proteins that were most prominently exposed to the host’s immune system [27]. We found that the biological processes enriched in DEGs obtained from the integrated data were mostly similar to those enriched in this study, indicating the robustness and scalability of the results.

**Table 1:** GO enrichment analysis of DGs in four different stages based on GEM and CNDM

Gene Ontology Consortium	
enriched biological process	enriched *p* value
type I interferon signaling pathway (GO:0060337)	5.56601E-09
defense response to virus (GO:0051607)	1.93858E-06
response to virus (GO:0009615)	4.1375E-06
protein targeting to ER (GO:0045047)	8.25505E-05
apoptotic process (GO:0006915)	0.002218377
response to type I interferon (GO:0034340)	0.009778615
response to interferon-beta (GO:0035456)	0.010751479
response to interferon-alpha (GO:0035455)	0.010751479
negative regulation of type I interferon-mediated signaling pathway (GO:0060339)	0.019466596
innate immune response (GO:0045087)	0.020208104
positive regulation of interleukin-10 production (GO:0032733)	0.039519859

**Table 2:** KEGG pathway analysis of DGs in four different stages based on GEM and CNDM

KEGG	
enriched biological process	enriched *p* value
Protein export(hsă0)	0.000498876
Coronavirus disease—COVID-19 (hsa05171)	0.044506896

### CCSN reveals network structure and dynamics on a single-cell basis

**Fig. 4:**
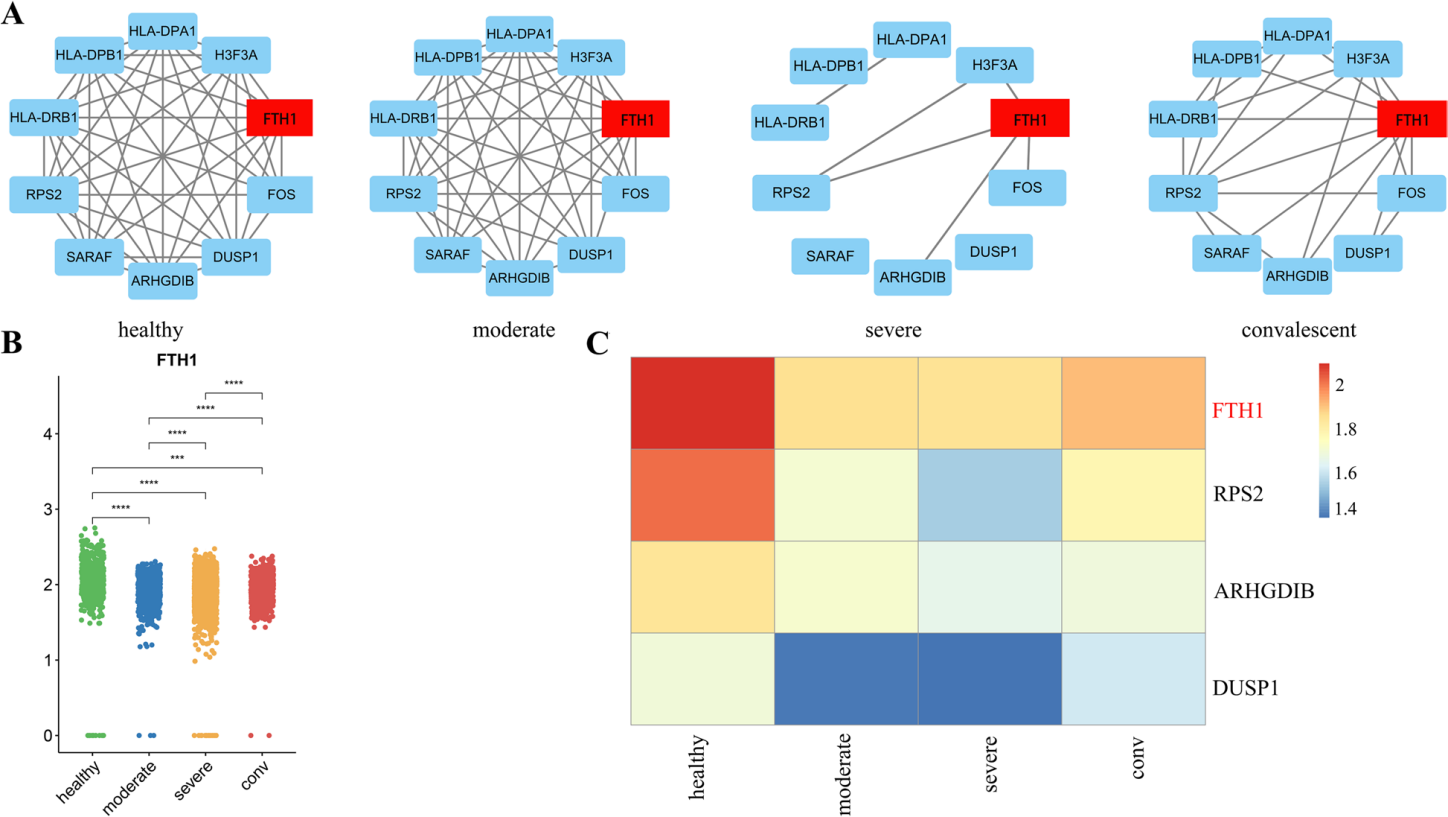
Illustration of network analyses of HAR000150 dataset based on CCSN method. **A** CCSNs of B cells with the 10 DDGs that were selected. The edge between two genes means the direct dependency of genes. **B** The conditional network degree level of the FTH1 at four conditions. Conditions are shown in different colors. All differences with *P* < 0.05 are indicated. **P < 0.01; ***P < 0.001; using student's test. **C** Heatmap of mean expression values of 4 DDGs in B cells under COVID-19 infection. Rows represent four different stages of B cells, columns represent genes, and different colors represent the expression values of different genes at different stages

According to the above research, the CCSN method can identify more differential genes, so the CCSN method has more advantages than the traditional gene expression method. We selected 10 genes from the DDGs of B cells. Figure 4A illustrates the partial CCSNs of the 10 DDGs. We could see the network topology changes dynamically at different stages. The associations between these genes were strongest in healthy controls. When infected with SARS-COV-2, the associations between these genes weakened in the moderate and became more weaker in the severe, and then gradually strengthened again in the convalescent. From Fig. 4A, we could see the association between FTH1 and other genes was the strongest in all four stages. The conditional network degree levels of FTH1 in the four stages were shown in Fig. 4B. Hence, FTH1 might play an important role in these four stages from network perspective. Therefore, FTH1 might be a hub gene due to the drastic network rewiring during the COVID-19 development.

The expression levels of these DDGs involved in the development of COVID-19 were different in the four stages. It could be seen that the expression levels of FTH1, RPS2, ARHGDIB and DUSP1 were significantly down-regulated during infection with SARS-COV-2 and up-regulated during the convalescent (Fig. 4C). These findings suggested that these genes encode proteins, which might be involved in viral interactions with the host and host immune responses and could reflect the severity of the disease. FTH1 was a hub gene in the network, and encoded the heavy subunit of ferritin-the major intracellular iron storage protein in prokaryotes and eukaryotes. Excess iron or iron repletion favors growth of many viruses [28]. Many of the important SARS-CoV-2 regulatory and functional proteins use iron [29]. Excess iron can also induce fibrin polymerization [30, 31] and induce a pro-coagulant state [32]. Previous literature reported occurrence of coagulopathy among severe COVID-19 patients [33, 34]. Kaushal et al. [35] found higher ferritin levels were found in COVID-19 patients [SMD -0.889 (95% C.I. -1.201, -0.577), I2 = 85%] compared to controls (COVID-19 negative), and higher ferritin levels were also found in severe COVID-19 patients compared to moderate COVID-19 patients [SMD 0.882 (0.738, 1.026), I2 = 85%]. Simultaneously, high serum ferritin level was associated with more severe disease and negative/poor outcome in COVID-19. Therefore, serum ferritin level could serve as an important predictive biomarker in COVID-19 management and in triage. And ferritin was a known inflammatory biomarker in COVID-19. Thus, FTH1 was a potential network marker in COVID-19.

Next, we further studied the expression of FTH1 at different stages of B cells. The expression of FTH1 was highest in healthy controls, lowest in severe, and increased again during convalescent. Although the expression level of FTH1 in the convalescent did not return to the healthy level in time, its expression level was also significantly higher than that in moderate and severe. Therefore, FTH1 may be a potential network marker for SARS-COV-2 infection, which needed to be further studied in the future.

Analysis of 32 common HLA alleles at four loci revealed a significant association between HLA-DRB1*09:01 and severe COVID-19, which indicate a potential role for HLA in predisposition to severe COVID-19 [36]. Different alleles of HLA-DPA1 are associated with the severity of covid-19. For example, HLA-DPA1*01:03 is associated with improved clinical outcomes of covid-19 [37], while HLA-DPA1*02:02 is associated with reduced antibody response after covid-19 vaccination [38]. HLA-DPB1*04:01 is associated with an increased risk of covid-19 double pneumonia [39], while HLA-DPB1*04:02 is associated with improved clinical outcomes of covid-19 [40]. HLA-DRB1*13:02 is associated with an increased risk of covid-19 symptoms, while HLA-DRB1*15:01 is associated with an enhanced T-cell response after covid-19 vaccination [41]. Notably, the mitogen-activated protein kinase (MAPK) pathway (i.e., FOS, JUN, JUNB, and DUSP1) was greatly suppressed in all recovered patients compared with that in the HCs, which suggested that inhibition of the MAPK signaling pathway is a recovery sign of COVID-19 patient [42]. The expression level of DUSP1 is particularly downregulated in the nasopharyngeal swabs and lung tissues of COVID-19 patients. This downregulation of DUSP1 could be the mechanism regulating the enhanced activation of MAPK pathway as well as the reported steroid resistance in SARS-CoV-2 infection [43]. The RPS2 gene is the gene that encodes the 40S ribosomal protein S2 [44]. SARS-CoV-2 nonstructural protein 1 (Nsp1), also known as host shutdown factor, can suppress host innate immune function. It has been recently found to bind to the 40S subunit of the human ribosomal complex and insert its C-terminal domain into the mRNA channel, thereby interfering with mRNA binding and impede the translation process of proteins [45, 46].

To better demonstrate the validity and generalizability of our results, we validated our findings on the COVID-19 dataset GSE155673. For details, see the Supplementary Note 4.

### ‘Dark’ genes analyses

If a gene had a significant difference between case and control samples not in a gene expression level but in a conditional network degree level, we called this gene ‘dark’ gene [17]. Downstream analysis of ‘dark’ genes mainly included prognostic analysis (Supplementary Note 5), protein level validation, enrichment analysis, cell–cell communication analysis and pathway analysis.

#### ‘Dark’ genes revealed by CNDM

In the field of bioinformatics, DEGs played an important role in finding new biomarkers, however some non-DEGs might also be involved in the biological process of disease. DEGs were obtained using traditional differential analysis methods, and we could also find DDGs at the network level based on CCSN methods. If a gene had a significant difference between case and control samples not in a gene expression level but in a conditional network degree level, we called this gene ‘dark’ gene [17]. These genes had no significantly differential changes in terms of gene expression, and thus could not be found by traditional differential analyses. We found a total of 287 ‘dark’ genes (Supplementary Table S1 and Fig. 2). Next we validated our findings on the COVID-19 dataset GSE155673. For details, see the Supplementary Note 4 and Supplementary Table S4.

**Fig. 5:**
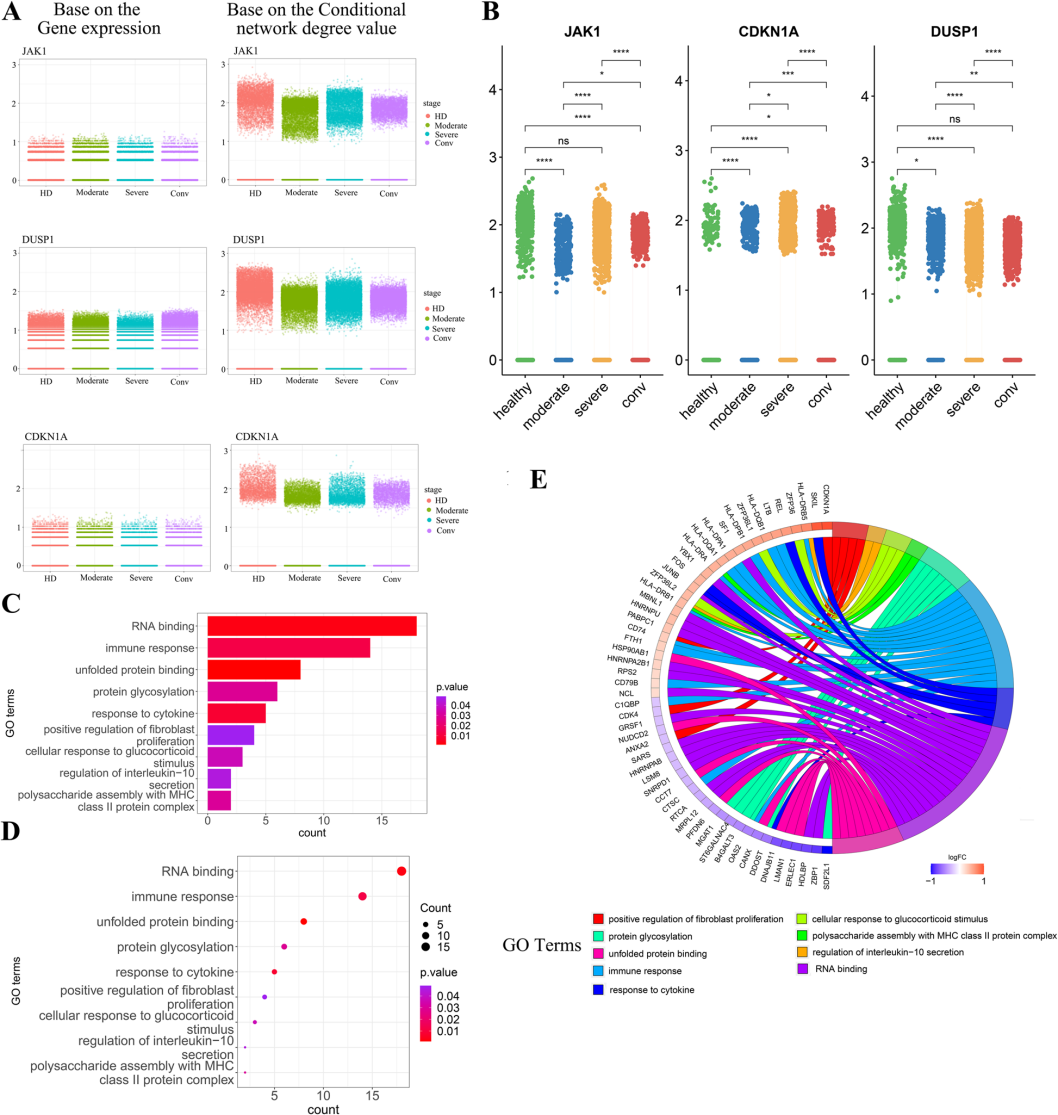
The differences of gene expression level and network degree level of some ‘dark’ genes in four different stages, and the validation and analysis of ‘dark’ genes. **A** Differences of the average gene expression value (left) and average network degree value (right) of JAK1, DUSP1 and CDKN1A at different stages. **B** The conditional network degree level of the JAK1, CDKN1A and DUSP1 at four conditions. Conditions are shown in different colors. All differences with *P* < 0.05 are indicated. **P < 0.01; ***P < 0.001; using student's test. **C** Bar plot of enriched GO terms. **D** Dot plot of enriched GO terms. **E** The GO chord plot shows the 54 ‘dark’ genes and the 9 enriched GO terms

Here we show the expression of some 'dark' genes in all 'dark' genes based on the gene expression level and the conditional network degree level, respectively. Figure 5A shows that there were not significant differences at gene expression levels for the ‘dark’ genes JAK1, DUSP1, CDKN1A, but there are significant differences at network degree levels (Fig. 5B).

JAK1 is an important gene in the JAK/STAT signaling pathway, which is one of the critical cellular signaling pathways involved in the inflammatory response [47]. In COVID-19 infection, an increase in the severity of the disease may be associated with an inflammatory state. Overwhelming inflammatory reactions contribute to respiratory distress in patients with COVID-19 [48]. Some literature suggests the treatment of patients with COVID-19 can be performed by JAK1/2 inhibitors [49].

The pathology of SARS-COV-2 is partly driven by a storm of cytokines, most of which are regulated by MAPK and NF-kB signaling pathways. The protein encoded by the ‘dark’ gene DUSP1 is a dual-specificity phosphatase 1 (DUSP1). DUSP1 exerts its anti-inflammatory effects through the dephosphorylation of p38 MAPKs, then reduces the pathway and downregulates the production of TNF-α, IL-1β, and IL-1A. DUSP1 inhibits MAPK signaling and pro-inflammatory cytokine production. So targeting DUSP1 to modulate MAPK and NF-κB pathways could constitute an attractive approach for the suppression of exaggerated inflammatory responses during COVID-19 infection [50].

The ‘dark’ gene CDKN1A is one of the targets associated with cell proliferation and cell cycle, and is also the promising target on which berberine may act to regulate immune responses, inflammatory processes, and cell activities against COVID-19 and SARS infection [51]. Expression of the senescence markers CDKN1A is significantly increased in epithelial ciliated and club cells from patients with severe COVID-19 compared with those with moderate disease and with healthy control subjects, suggesting that lung cell senescence induction coincided with virus detection [52].

Therefore, some ‘dark’ genes had been found to be associated with COVID-19 and some other diseases of the lungs, and inflammation, which indicated these ‘dark’ genes play an important role in the complications caused by COVID-19.

#### ‘Dark’ genes validated at protein level

We used the proteomics data to show that some ‘dark’ genes were significantly expressed at the protein level and could also play functional roles during the development process of COVID-19. The proteomic data was obtained from lung tissue studies from patients with COVID-19, and the bronchoalveolar lavage fluid (BALF) from five COVID-19 patients and four non-COVID-19 patients [53, 54]. The corresponding results showed that a quantitative proteomic approach combined with bioinformatics analysis was carried out to detect proteomic changes in the SARS-CoV-2-infected human lung tissues. A total of 38 proteins [53] were identified to be differentially expressed (BH adjusted *p* < 0.05 and log2COVID-19/Control > 1 or < -1). Among them, 9 proteins were up-regulated (avg_log2FC > 0) and 29 proteins were down-regulated (avg_log2FC < 0) in response to SARS-CoV-2 infection. Based on a quantitative proteomic strategy to investigate the alterations of BALF proteome in COVID-19 patients compared with the non-COVID-19 controls, a total of 66 proteins corresponding to the ‘dark’ genes overlapped across all the patients were successfully quantified. 29 proteins corresponding to the ‘dark’ genes were differentially expressed (fold change > 2, *P* < 0.05), among which 28 proteins were down-regulated, and one protein were up-regulated [54]. Proteomic data obtained from the based lung tissue and BALF studies of COVID-19 patients demonstrates that some ‘dark’ genes were closely related to the development of COVID-19. Details of the proteins corresponding to the ‘dark’ genes were provided in Supplementary Table S3.

In addition, by significance test with hypergeometric distribution for the results of COVID-19, the probability of 67 proteins with high confidence at the differential expression level among 287 ‘dark’ genes (obtain from 5000 genes) was $1.77e - 24$. The results of hypergeometric test showed that the protein level of ‘dark’ genes is significantly higher than that of other genes. Therefore, the ‘dark’ genes might play an important role in the development of COVID-19 from the perspective of protein level.

#### ‘Dark’ genes GO enrichment analysis

As shown in Table 3, some ‘dark’ genes were enriched into the biological processes associated with COVID-19 (Supplementary Table S2), e.g. ‘positive regulation of fibroblast proliferation’ (GO:0048146), ‘regulation of interleukin-10 secretion’ (GO:2,001,179), ‘protein glycosylation’ (GO:0006486), ‘immune response’ (GO:0006955), ‘RNA binding’ (GO:0003723) and others in Gene ontology (Fig. 5C-E). Table 4 shows some ‘dark’ genes are enriched in the KEGG signaling pathway, e.g. ‘Apoptosis’ (hsa04210), ‘Antigen processing and presentation’ (hsa04612), ‘RNA degradation’ (hsa03018) and others. At the core of tissue remodeling in COVID-19 infected alveolar tissue may be fibroblasts. Fibroblasts in the lung interstitium are the common cells, producing extracellular matrix and active during the injury [55]. COVID-19 virus may also infect the fibroblasts, which may cause proliferation of fibroblasts and extracellular matrix over production [56]. A unique feature of the cytokine storm in COVID-19 is the dramatic elevation of interleukin 10 (IL-10), which is regarded as a negative feedback mechanism to suppress inflammation. Dramatic early proinflammatory IL-10 elevation may play a pathological role in COVID-19 severity [57]. The SARS-CoV-2 uses its highly glycosylated spike (S) glycoproteins to bind to the cell surface receptor angiotensin-converting enzyme 2 (ACE2) glycoprotein and facilitate host cell entry [58]. SARS-CoV-2 is an RNA virus whose success as a pathogen relies on its abilities to repurpose host RNA-binding proteins (RBPs) and to evade antiviral RBPs [59]. These results suggested that some ‘dark’ genes were involved in the immune response to SARS-COV-2.

**Table 3:** GO enrichment analysis of ‘dark’ genes

Gene Ontology Consortium	
enriched biological process	enriched *p* value
positive regulation of fibroblast proliferation (GO:0048146)	0.048722947
regulation of interleukin-10 secretion (GO:2,001,179)	0.044871716
cellular response to glucocorticoid stimulus (GO:0071385)	0.036463359
polysaccharide assembly with MHC class II protein complex (GO:0002506)	0.030141888
protein glycosylation (GO:0006486)	0.02902154
immune response (GO:0006955)	0.012340031
response to cytokine (GO:0034097)	0.007946145
RNA binding (GO:0003723)	0.002965349
unfolded protein binding (GO:0051082)	0.001168546

**Table 4:** KEGG pathway analysis of ‘dark’ genes

KEGG	
enriched biological process	enriched *p* value
Antigen processing and presentation(hsa04612)	1.58E-06
Influenza A(hsa05164)	1.64703E-05
RNA degradation(hsa03018)	0.000636635
Apoptosis(hsa04210)	0.013345032
Measles(hsa05162)	0.044979984

#### Cell–cell communication analysis

**Fig. 6:**
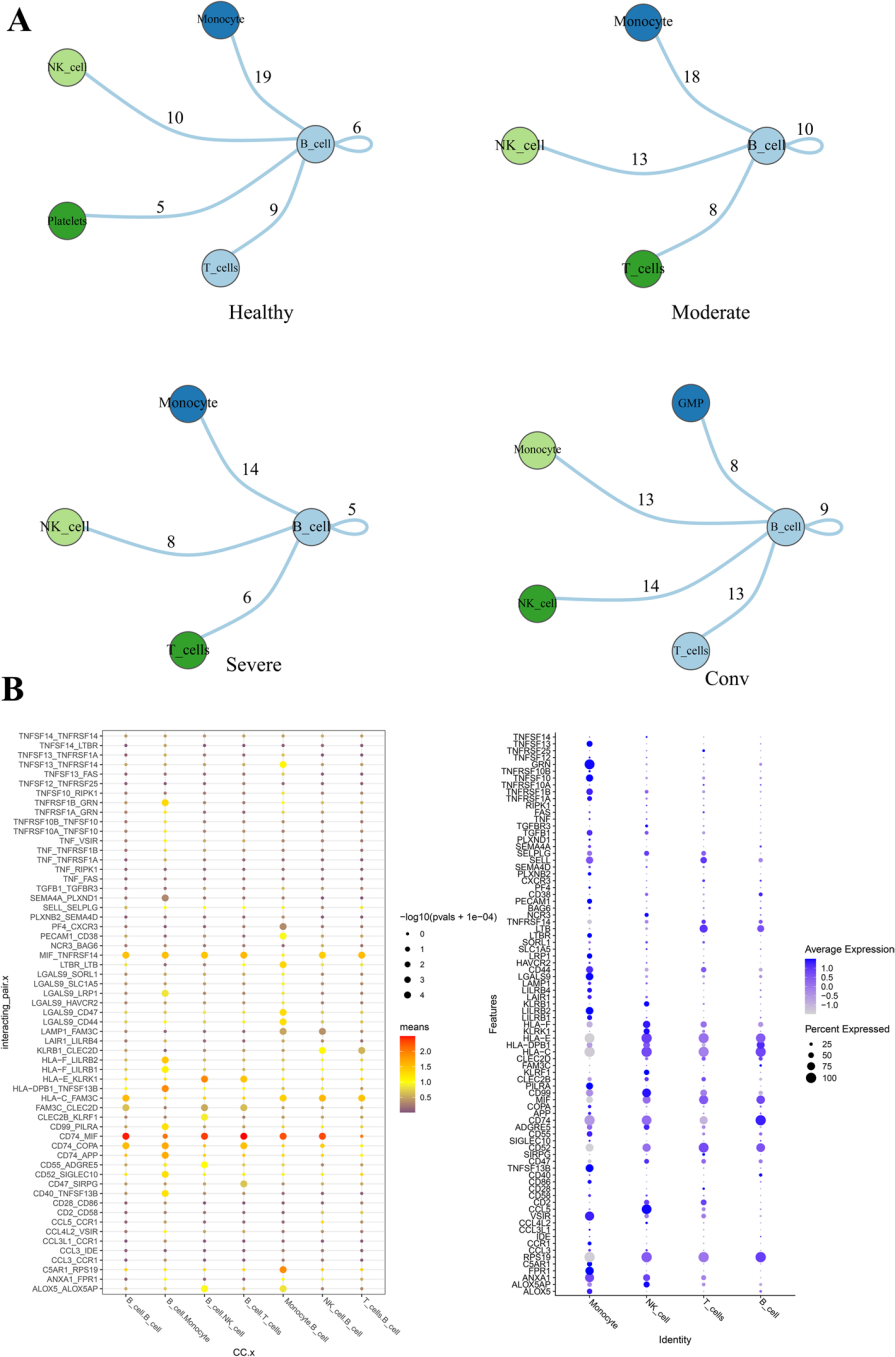
Differential interactions among B cells and other cell types in different stages. **A** The connectivity map shows the strength of interactions between B cells and other cell types in colored shells. The numbers represent the frequency of communication. Different colored circles represent different cell types. **B** The B_cells-centric molecular interactions of peripheral immune cells in severe-stage COVID-19 patient. *P*-values are represented by the size of the circle, and the ruler is shown on the right side of the graph. The color represents the average expression level of these two genes in the two cell clusters, and the darker the color, the higher the expression. The size of the bubble represents the -log10 value of the *P* value, and the larger the bubble, the more significant it is

Cell–cell communication analysis was performed by using CellPhoneDB (www.cellphonedb.org), which is a publicly available repository of curated receptors and ligands and their interactions. Single-cell transcriptome data of cells annotates as Monocyte, NK cells, T cells, platelets, GMP and B cells were input into CellPhoneDB for cell–cell interaction analysis. Enriched receptor-ligand interactions between two cell types were derived based on the expression of a receptor by one cell type and the expression of the corresponding ligand by another cell type [60]. The B cells of the immune system in COVID-19 patients were fully fighting the SARS-CoV-2 virus. The key characteristics of B cells were effective at neutralizing, or inactivating, the SARS-CoV-2 virus and related coronaviruses [24]. So we focused on the role of B cells in COVID-19 and conducted the interaction between B cells and other cell types. Enriched ligand-receptor interactions between two cell states are obtained by utilizing a receptor expressed by one cell state and a ligand expressed by another cell state. We calculate the percentage of cells expressing each gene and the mean expression level for genes within the cluster. Expression levels of ligands and receptors in each cell state are taken into consideration, and empirical shuffling is employed to identify ligand-receptor pairs that exhibit notable specificity towards a particular cell state. We selected the receptor ligand and the genes common in our data as the genes in our analysis. Figure 6A showed the number of communication frequencies between the B cell and other cell types, namely interactions. Especially in the severe stage, the interaction between B cells and other cell types was significantly weakened. The main reason might be that the B cells enhanced the COVID-19 severity by aggravating inflammation, thus resulting in a dysregulated of immune response to SARS-CoV-2 [25, 61]. We therefore reasoned that the severe stage was an important stage for B cells to function. Simultaneously, given that B cells in the severe stage might be involved in the regulation of a variety of immune cell types [62], we would investigate the potential interactions between B cells and other cell types in severe stage. Interestingly, the interaction between B cells and Monocyte was the most obvious. In respiratory infections, monocytes in the lungs developed into macrophages that fight viruses and bacteria. However, a certain type of macrophages might also promote severe inflammation and infection. The most interacting pairing of B cells with other cell types was CD74_MIF (Fig. 6B), which played an important role in the process of inflammatory diseases. We found that MIF was DEG and CD74 was both DDG and ‘dark’ gene, which indicated that the combination of DEGs and DDGs could be better used to study COVID-19 in depth. MIF was a truly pleiotropic inflammatory cytokine that was expressed by a variety of cells, and was a critical upstream mediator of innate immunity [63]. CD74 was the main receptor for MIF. CD74 was the invariant chain of the MHC class II and played an important role in antigen presentation. MIF and CD74 plays an important role in the recovery of tissue injury and acute lung injury [63]. During acute injury such as viral infection, type I cells release MIF. Extracellular MIF binds to CD74 on adjacent type II epithelial cells, activating Akt and ERK pathways, resulting in cell proliferation and differentiation to restore the alveolar barrier [64]. In COVID-19 infection, the inflammatory response is one of the key factors in disease progression and severity. The interaction between CD74 and MIF may be involved in regulating the inflammatory response in COVID-19 patients, affecting the activation of immune cells and the release of inflammatory mediators. The combination of MIF and CD74 could also inhibit cell apoptosis, promote cell proliferation and alveolar epithelial cell repair, and at the same time play a role in reducing lung inflammation and acute lung injury. MIF and CD74 were emerging attractive targets for immune therapy [63]. Therefore, MIF and CD74 might become targets for COVID-19 treatment. Thus the requires further clinical validation to confirm our inferences. We also performed cell–cell communication analysis between B cells and other cell types and studied B_cells-centric molecular interactions of peripheral immune cells in healthy, convalescence and moderate COVID-19 patients, and details were shown in Supplementary Note 6.

#### The underlying signaling mechanisms revealed by ‘dark’ genes

**Fig. 7:**
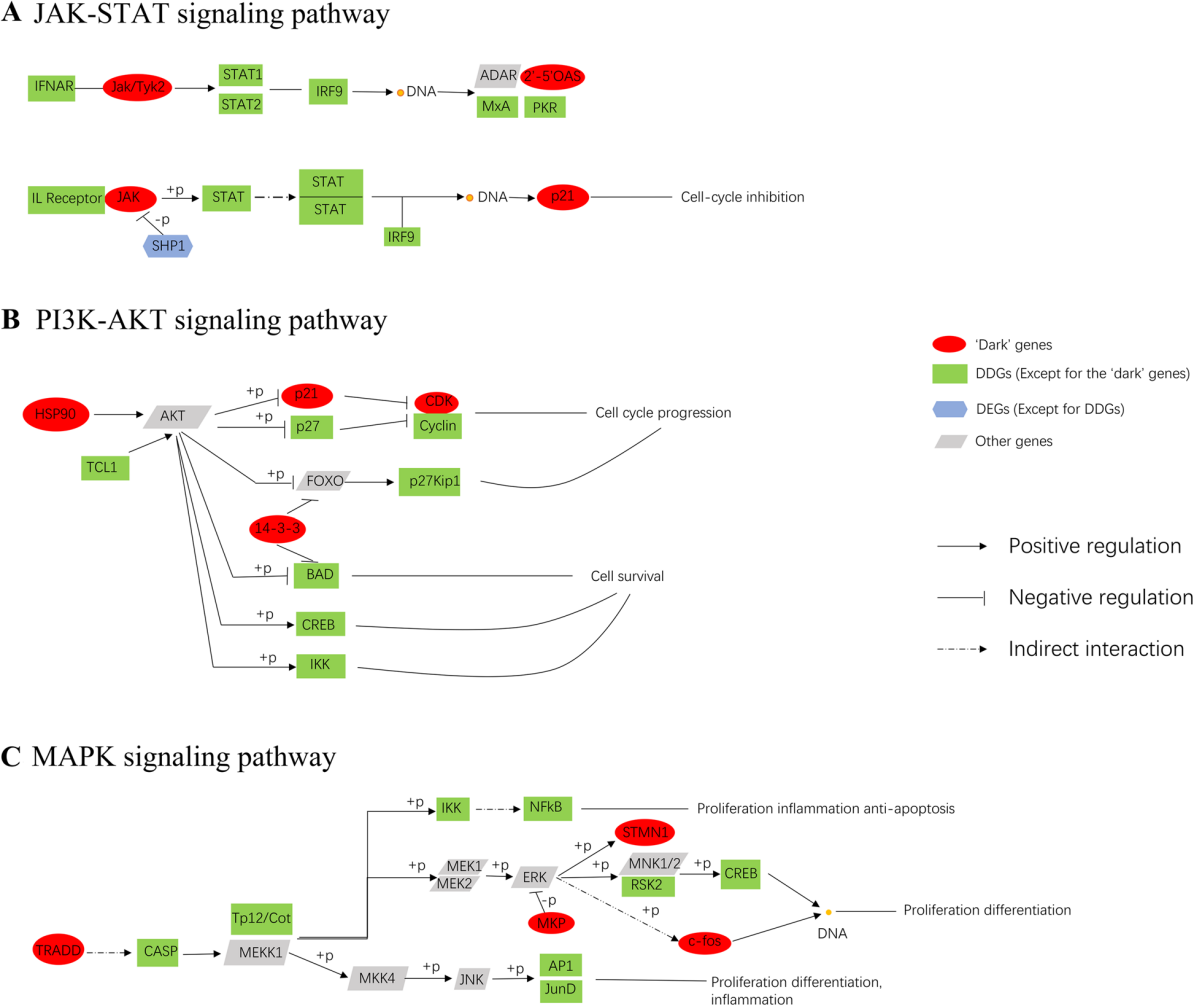
Potential mechanisms revealed by the functional analysis of ‘dark’ genes, DDGs and DEGs. **A** Regulation of related ‘dark’ genes, DEGs and DDGs in the JAK-STAT signaling pathway. **B** Regulation of related ‘dark’ genes, DEGs and DDGs in the PI3K-AKT signaling pathway. **C** Regulation of related ‘dark’ genes, DEGs and DDGs in the MAPK signaling pathway

The ‘dark’ genes JAK1 and OAS2 encoded the proteins JAK and 2’-5’OAS (2’-5’-Oligoadenylate synthetase) in the JAK-STAT signaling pathway in the Coronavirus disease—COVID-19 [65–67], respectively. The interferon-α/β receptor IFNAR, co-encoded by the DDGs IFNAR1 and IFNAR2, activated the JAK-STAT signaling pathway, which led to activation of the JAK kinase encoded by the ‘dark’ gene JAK1, followed by phosphorylation of STAT1 and STAT2 proteins. Phosphorylated STATs dissociated from the receptor heterodimer and bound to IRF9 (Interferon Regulatory Factor-9), a member of the IRF family, and transported it to the nucleus, where it directly bound to DNA and activated the protein MxA (myxovirus-resistance A), 2’-5’ OAS and PKR (Protein kinase R) (Fig. 7A).

To resist SARS-CoV-2, the protein 2’-5’OAS regulates the RNaseL pathway, the protein PKR activates both exerted antiviral functions, and they were all associated with viral escape [68]. Since the protein MxA encoded by DDGs Mx1 and Mx2 had antiviral activity, MxA might also be activated by SARS-CoV-2 to exert antiviral functions. See Supplementary Note 7 for specific details on their antiviral function.

By consulting the literature, we found that several drugs (Ruxolitinib, Baricitinib, Tofacitinib and Upadacitinib) could inhibit the activity of JAK and block the signaling of multiple interleukins, thus reducing inflammatory cascade, knocking down the hyper inflammation, decreasing the lung impairment and restoring the PaO2/FiO2 ratio in COVID-19 patients [69, 70].

To further clarify the pathway associations of ‘dark’ genes and their differential genes in B cells, in this study, we focused on the analysis of the KEGG pathway on the JAK-STAT signaling pathway and PI3K-AKT signaling pathway that were most relevant to the progression of COVID-19 (Fig. 7A, B). The protein p21 encoded by the ‘dark’ gene CDKN1A was involved in the JAK-STAT signaling pathway and the PI3K-AKT signaling pathway, which was a key regulator in common with these two pathways.Thus, p21 played an important regulatory role in the transmission mechanism of COVID-19. The up-regulation of p21 expression could inhibit the cell cycle, and the down-regulation of p21 expression promoted the process of cell cycle. Meanwhile, elevated expression levels of proteins encoded by some DDGs are directly correlated with the severity of COVID-19 patients. See Supplementary Note 7 for details on how these two pathways function.

The proteins encoded by the ‘dark’ genes TRADD, FOS and DDGs RELB, JUN, IKBKG are involved in the MAPK signaling pathway, which is a potential signaling pathway for the spread and development of COVID-19(Fig. 7C). For details, see Supplementary Note 7.

B cell growth and proliferation were tightly regulated by signaling through the B cell receptor (BCR) and by other membrane bound receptors responding to different cytokines [71]. BCR guided and controlled every stage of B cell life. Upon BCR antigen binding, this resulted in the activation of NF-kB, PI3K and MAPK signaling pathways [72]. The PI3K signaling pathway had been shown to play a crucial role in B cell activation, differentiation and survival [71], while PI3K signaling was a major regulator of B-cell metabolism and redox signaling [72]. PI3K signaling and MAPK signaling were initiated downstream of the BCR and affect B cell growth and activation. B cells were activated after encountering SARS-COV-2, and part of them becomes highly efficient and short-lived plasma cells, which secreted antibodies to clear SARS-COV-2. The other part became long-lived memory cells, with a secondary response occurring to immediately eliminate the re-invading SARS-COV-2. SARS-CoV-2 infection could alter the BCR signaling through inhibiting the early activation of naive and memory B cells, thus leading to immunodeficiency in the recovered patients.

## Discussion

With the development of single-cell sequencing technology, new algorithms have provided an unprecedented opportunity to identify gene associations/networks at the single-cell resolution level [18]. Li et al. presented a new method to construct a CCSN for each single cell from scRNA-seq data (i.e. one network for one cell). Each CCSN could be viewed as the transformation from less ‘reliable’ gene expression to more ‘reliable’ gene–gene associations in a cell. By applying the CCSN method to the single-cell dataset HRA000150 of COVID-19, we found that the associations between some genes were strong in the healthy controls, but significantly weakened in the diseased phase, especially in the severe, and then strengthened again in the convalescent. These results suggested that the drastic network rearrangement process might occur at the severe, further illustrating the possibility that the severe may be a key stage in these networks, and network markers in severe cells could be used as a representative indicator of potential infection and treatment prognosis of COVID-19. We visualized the CCSNs at different stages, and then ranked 10 DDGs according to the degree of association of genes at each stage. The most associated gene in the CCSN is FTH1. The hub gene FTH1 encoded the heavy subunit of ferritin. Ferritin was a known inflammatory biomarker in COVID-19, and serum ferritin levels could serve as an important predictive biomarker in the management and triage of COVID-19. Hence the FTH1 might be a potential network marker for COVID-19, which needed to be further studied in future. Next, we performed GO and KEGG enrichment analysis on DGs, which verified the important role of the DDGs obtained based on CNDM related to COVID-19. To enhance the robustness of the research findings, we integrated three datasets, namely HRA000150, HRA001149, and GSE155673, into the analysis. The integrated data was subjected to differential expression analysis to obtain DEGs, followed by enrichment analysis. We compared our study with other published studies with details in the Supplementary Table S5.

Some ‘dark’ genes were related to COVID-19. Prognostic analysis and protein level validation of ‘dark’ genes revealed that ‘dark’ genes had important biological significance and played an important functional role in the progression of COVID-19. Besides, by cell–cell communication between B cells and other cell types, we found B cells play a crucial part in the severe stage, while the receptor ligand that B cells interact most with other cell types was CD74_MIF, of which CD74 was a ‘dark’ gene. MIF and CD74 were emerging as attractive candidates for immunotherapy force targets.

In order to reveal the functional underlying mechanisms of ‘dark’ genes, DDGs and DEGs, this study focused the KEGG pathway analysis on three pathways closely related to COVID-19: JAK/STAT signaling pathway, PI3-ATK signaling pathway and MAPK signaling pathway. Interestingly, MAPK signaling pathway was a potential signaling pathway for the spread and development of COVID-19. The proteins encoded by these ‘dark’ genes complement some of the missing links in Coronavirus disease—COVID-19. These results also showed that the combination of CNDM and GEM methods was helpful to discover new signaling pathway involved factors or biomarkers.

There are also some limitations of this study, which are detailed below. Firstly, the relatively small sample size was the limitation of study. Therefore, future studies with longitudinal samples from more patients with COVID-19 would lead to a better understanding of the pathogenic mechanism of COVID-19. Secondly, the therapeutic targets of COVID-19 we obtained are almost potential, so our study is a preliminary conclusion. Further clinical validation is therefore required to confirm our inferences. Finally, the samples collected from PBMC and BALF represent different cell populations and microenvironments, which limited the generalizability of the findings to the study.

## Conclusions

In this study, CCSN was constructed for each cell, and B cells were extracted for subsequent analysis. From a network perspective, single-cell association analysis not only laid the foundation for future characterization of the complex, dynamic immune responses to SARS-CoV-2 infection, but also helped to find potential network markers and signaling pathways for COVID-19, providing substantial value to further clarify the involvement of B cells in COVID-19.

### Supplementary Information

**Additional file 1:** **Table S1A.** Differential expression genes obtained from healthy and moderate comparative analysis based on GEM. **Table S1B.** Differential expression genes obtained from healthy and severe comparative analysis based on GEM. **Table S1C.** Differential expression genes obtained from healthy and convalescent comparative analysis based on GEM. **Table S1D.** Differential degree genes obtained from healthy and moderate comparative analysis based on CNDM. **Table S1E.** Differential degree genes obtained from healthy and severe comparative analysis based on CNDM. **Table S1F.** Differential degree genes obtained from healthy and convalescent comparative analysis based on CNDM. **Table S1G.** DGs. **Table S1H.** 'dark' genes. **Table S1I.** DDGs.**Table S1J.** DEGs. **Supplementary Note 1.** Construct of CCSN and obtain CNDM from CCSN. **Supplementary Note 2.** Network-based cell clustering, gene dimension-reduction analysis and cell counts. **Supplementary Note 3.** ‘Dark’ genes revealed by CNDM. **Supplementary Note 4.** Validation of experimental results based on the COVID-19 single-cell sequencing dataset GSE155673. **Supplementary Note 5.** The prognosis analysis of ‘dark’ genes. **Supplementary Note 6.** Cell-cell communication analysis. **Supplementary Note 7.** The underlying signaling mechanisms revealed by ‘dark’ genes.

## Data Availability

Publicly available datasets were analyzed in this study. Data supporting the findings of this study are available in the Genome Sequence Archive of the BIG Data Center, Beijing Institute of Genomics (BIG), Chinese Academy of Sciences.at http://bigd.big.ac.cn/gsa-human, reference number HRA000150 and HRA001149, and Gene Expression Omnibus (GEO) (accession number GSE155673). The code used for this paper is available on GitHub (https://github.com/HLQsmile/code).

## Abbreviations

CNDM: Conditional network degree matrix
GEM: Gene expression matrix
DDG: Differential degree genes
SARS-CoV-2: Severe acute respiratory syndrome coronavirus 2
COVID-19: Coronavirus disease 2019
CSN: Cell-specific network
NDM: Network degree matrix
DEG: Differentially expressed genes
BIG: Beijing Institute of Genomics
IFN-I: Type I interferons
DUSP1: Dual-specificity phosphatase 1
BALF: Bronchoalveolar lavage fluid
ACE2: Angiotensin-converting enzyme 2
RBP: RNA-binding protein
IRF9: Interferon Regulatory Factor-9
PKR: Protein kinase R
2’-5’ OAS: 2’-5’ -Oligoadenylate synthetase
MAPK: Mitogen-activated protein kinase
