# Supplementary material for: Potential network markers and signaling pathways for B cells of COVID-19 based on single-cell condition-specific networks
Source: BMC Genomics. 2023 Oct 18;24:619. doi: 10.1186/s12864-023-09719-1 (PMC10583333; doi:10.1186/s12864-023-09719-1)
Supplement: Supplementary file 1 — Additional file 1: Table S1A. Differential expression genes obtained from healthy and moderate comparative analysis based on GEM. Table S1B. Differential expression genes obtained from healthy and severe comparative analysis based on GEM. Table S1C. Differential expression genes obtained from healthy and convalescent comparative analysis based on GEM. Table S1D. Differential degree genes obtained from healthy and moderate comparative analysis based on CNDM. Table S1E. Differential degree genes obtained from healthy and severe comparative analysis based on CNDM. Table S1F. Differential degree genes obtained from healthy and convalescent comparative analysis based on CNDM. Table S1G. DGs. Table S1H. 'dark' genes. Table S1I. DDGs.Table S1J. DEGs. Supplementary Note 1. Construct of CCSN and obtain CNDM from CCSN. Supplementary Note 2. Network-based cell clustering, gene dimension-reduction analysis and cell counts. Supplementary Note 3. ‘Dark’ genes revealed by CNDM. Supplementary Note 4. Validation of experimental results based on the COVID-19 single-cell sequencing dataset GSE155673. Supplementary Note 5. The prognosis analysis of ‘dark’ genes. Supplementary Note 6. Cell-cell communication analysis. Supplementary Note 7. The underlying signaling mechanisms revealed by ‘dark’ genes. [file 12864_2023_9719_MOESM1_ESM.zip › Revised supplementary information/Supplementary Material.docx]

**Potential network markers and signaling pathways for B cells of COVID-19 based on single-cell condition-specific networks**

**Supplementary Material**

**Ying Li1,2,†, Liqin Han1,2,†, Peiluan Li1,2,*, Jing Ge3, Yun Xue4 and Luonan Chen5,6,7,8***

1 School of Mathematics and Statistics, Henan University of Science and Technology, Luoyang, 471000, China

2 Longmen Laboratory, Luoyang, Henan, 471003, China

3 Shanghai Immune Therapy Institute, Renji Hospital, Shanghai Jiao Tong University School of Medicine, 200032, Shanghai, China

4 College of Medical Technology and Engineering, Henan University of Science and Technology, Luoyang, 471023, China

5 Key Laboratory of Systems Biology, Institute of Biochemistry and Cell Biology, Center for Excellence in Molecular Cell Science, Chinese Academy of Sciences, Shanghai, 201100, China

6 Key Laboratory of Systems Health Science of Zhejiang Province, Hangzhou Institute for Advanced Study, University of Chinese Academy of Sciences, Hangzhou, 310000, China

7 School of Life Science and Technology, ShanghaiTech University, Shanghai, 201100, China

8 West China Biomedical Big Data Center, Med-X center for informatics, West China Hospital, Sichuan University, Chengdu 610041, China

**†** These authors contributed equally to this paper as the first authors.

**Contents**

[Supplementary Note 1: Construct of CCSN and obtain CNDM from CCSN 1](#_Toc135764706)

[Supplementary Note 2: Network-based cell clustering, gene dimension-reduction analysis and cell counts 3](#_Toc135764707)

[Supplementary Note 3: ‘Dark’ genes revealed by CNDM 5](#_Toc135764708)

[Supplementary Note 4: Validation of experimental results based on the COVID-19 single-cell sequencing dataset GSE155673 7](#_Toc135764709)

[Supplementary Note 5: The prognosis analysis of ‘dark’ genes 9](#_Toc135764710)

[Supplementary Note 6: Cell-cell communication analysis 11](#_Toc135764711)

[Supplementary Note 7: The underlying signaling mechanisms revealed by ‘dark’ genes 12](#_Toc135764712)

[References 16](#_Toc135764713)

#

# Supplementary Note 1: Construct of CCSN and obtain CNDM from CCSN

Assume thatandare two random variables, andis the third random variable. we construct a statistic (1)

(1)

to measure the conditional independence between geneand geneon the condition of genein cell . where 、 、 、 are the numbers of cells in neighborhoods 、、 and , respectively. The range of the statistic is -1 to 1. If , it means gene and gene are related in cell , and there is an edge. Otherwise, gene and gene are independent, and there is no edge. The CCSN can identify direct associations between a pair of genes in a cell by eliminating indirect associations between genes by selecting a small number of conditional genes.

The following steps were used to obtain the conditional genes. For a given cell k, we choose the toplargest ‘importance’ genes as the conditional genes. We assume that the conditional gene set is, and CCSN is obtained for cellgiven conditional gene.

The CCSNs of the cellon the condition of gene set are . Then, we use

(3)

to represent the degrees of gene–gene interaction network of cell k, where for is theelement of the matrix .

We transform Equation (3) to a conditional network degree vector based on the following transformation

(4)

Then, for , an matrix conditional network degree matrix (CNDM) is obtained.

with (5)

The matrix has the same dimension with the gene expression matrix (GEM), CNDM can reflect the gene–gene direct association in terms of interaction degrees. Moreover, after normalization, this CNDM matrix could be further analyzed by most traditional scRNA-seq methods for dimension reduction and clustering analysis.

The input and output of CCSN method are as described below.

Input: Gene expression matrix (FPKM/TPM/RPKM/count)

Significance level (e.g. 0.001, 0.01, 0.05 …).

Box size: The box size is the size of neighborhood in our algorithm. Default = 1.5. Users can change this parameter as well.

: the number of conditional genes. Default =1. Users can change this parameter as well.

Output: Conditional cell-specific network for each cell for a given conditional gene. (row= genes, column= cells)

Conditional network degree matrix (row = genes, column = cells).

# Supplementary Note 2: Network-based cell clustering, gene dimension-reduction analysis and cell counts


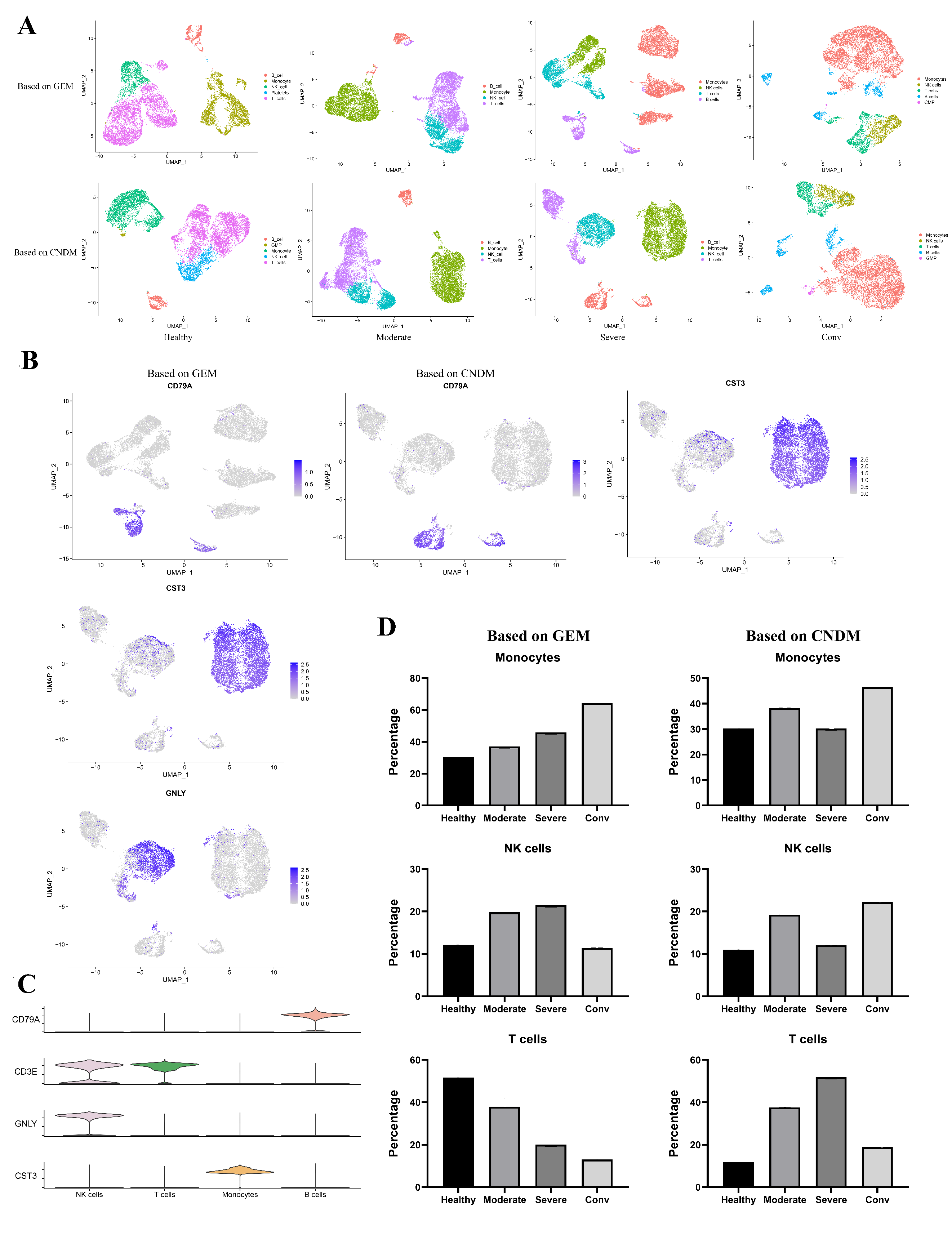


**Supplementary Figure S1.** **(A)** Comparison of cluster visualization for severe stages based on GEM and CNDM of the scRNA-seq dataset (HRA000150). UMAP projections of GEM and CCSN for healthy, moderate, severe and convalescent stages of dataset HRA000150. Each dot represents a single cell, colored according to cell type. **(B)** cell types annotation. Scatter plots show expression of canonical markers of severe stage cell types and the cells are colored according to conditional network degree levels. **(C)** Violin plot shows classical markers annotating cell types based on conditional network degree levels. **(D)** Bar plot representing the percentage of monocytes, NK cells and T cells among all cell types of each condition based on GEM and CNDM, respectively.

Based on GEM and CNDM at four stages, we performed clustering for cells, respectively (Fig.S1A). We obtained the transcriptomes of 5 major cell types in healthy and convalescence, and the transcriptomes of 4 major cell types in moderate and severe. According to the expression level of canonical markers CD79A, a total of 1862 B cells were identified based on GEM, a total of 1829 B cells were identified based on CNDM, and these B cells were extracted for subsequent detailed analyses (Fig.S1B, C). We calculated the relative percentage of the 4 cell types in the peripheral blood cells of each individual on the basis of GEM and CNDM (Fig.S1D). Based on CNDM, the relative percentages of NK cells and Monocytes increased in moderate, decreased in severe, and increased in convalescence, and the relative percentage of T cell cluster peaked in severe.

# Supplementary Note 3: ‘Dark’ genes revealed by CNDM


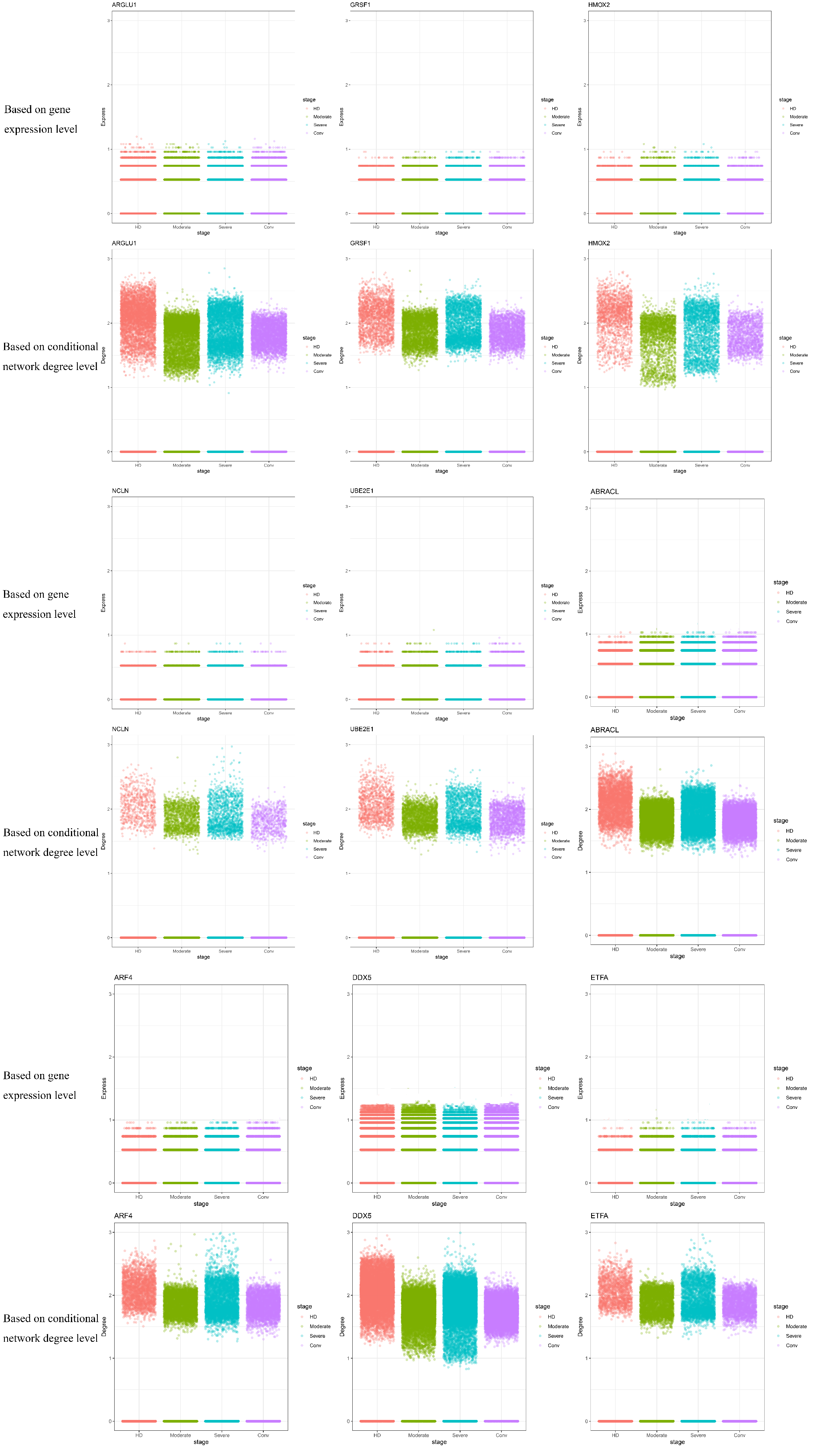


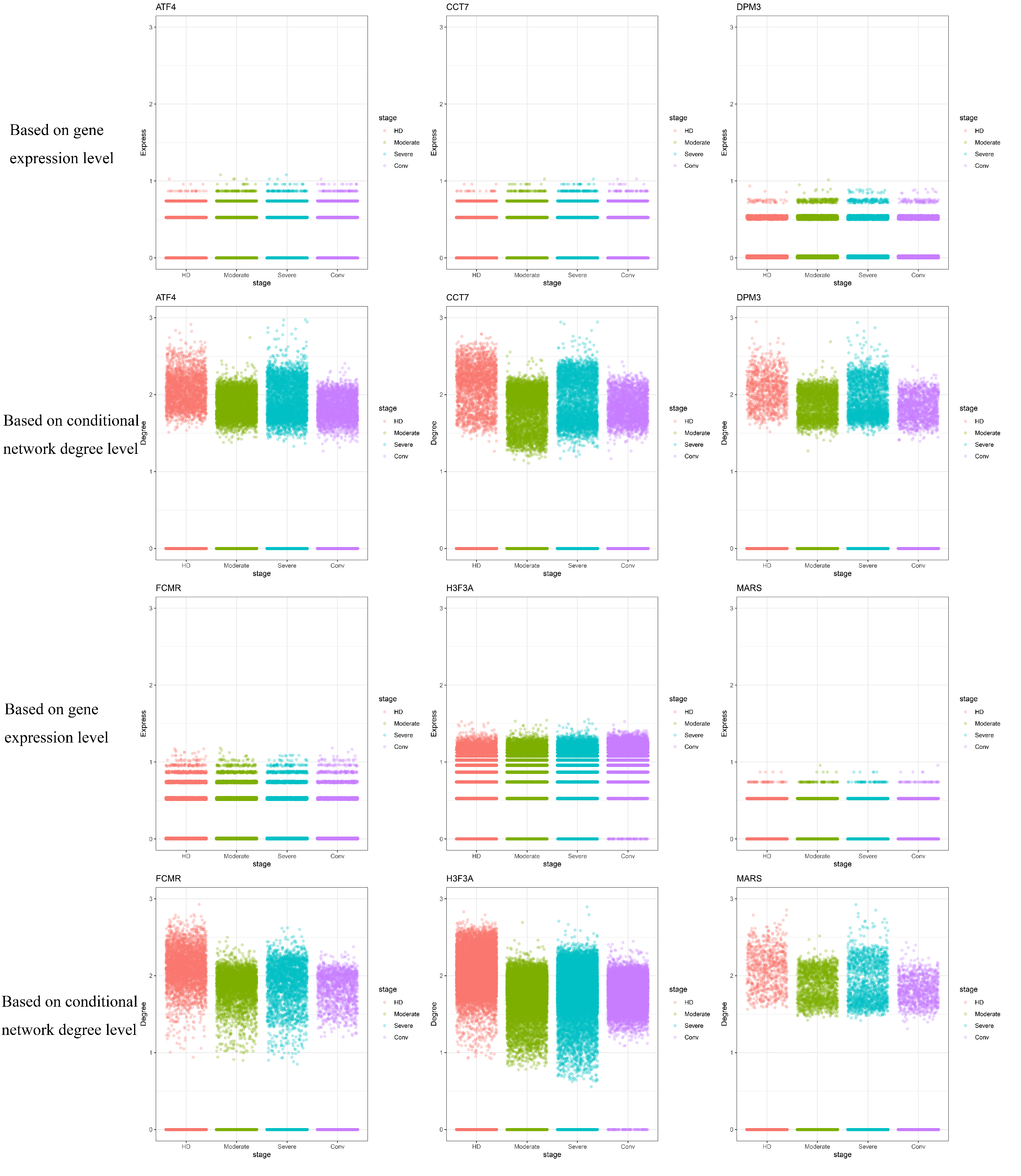


**Supplementary Figure S2.** The differences of gene expression level and conditional network degree level of some ‘dark’ genes in four different stages.

Fig. S2 shows that there were not significant differences at gene expression levels for some ‘dark’ genes, but there are significant differences at network degree levels.

The main role of DDX5 when hijacked by coronavirus is to positively modulate viral genome transcription and virus proliferation. Through direct binding to the SARS-COV helicase, DDX5 may act as a coactivator to enhance viral genome transcription and virus proliferation (2). Modulating ATF4 activity could moderate clinical complications of coronavirus disease 2019 (COVID-19) by cutting down GRP78 levels (3). Therefore, some ‘dark’ genes had been found to be associated with COVID-19, which indicated these ‘dark’ genes play an important role in COVID-19.

# Supplementary Note 4: Validation of experimental results based on the COVID-19 single-cell sequencing dataset GSE155673

We successfully validated our findings in the GSE155673 dataset. The data was produced by Arunachalam et al., a research group in the USA, in 2020 (Arunachalam et al. 2020). There were PBMC samples of seven patients with COVID-19 and five healthy controls. Three out of the seven patients showed moderate disease symptoms, and the rest showed severe symptoms according to the WHO classification.

We replicated our experimental design and analytical pipeline using this dataset and obtained results consistent with our initial study. Our findings in this dataset align with our initial observations, confirming the dynamic changes in network topology across different stages of infection (Fig.S3). Specifically, we observed that the associations between these genes were indeed strongest in healthy controls. Upon SARS-COV-2 infection, these associations weakened in the moderate cases and became significantly weaker in the severe cases. Furthermore, in the analysis of the dataset GSE155673, we identified 446 ‘dark’ genes, of which 67 overlapped with the previously identified ‘dark’ genes (Fig.S4). This additional validation strengthens our findings and provides further support for the presence of these genes in the context of COVID-19.

These validations further strengthen the reliability and generalizability of our findings.


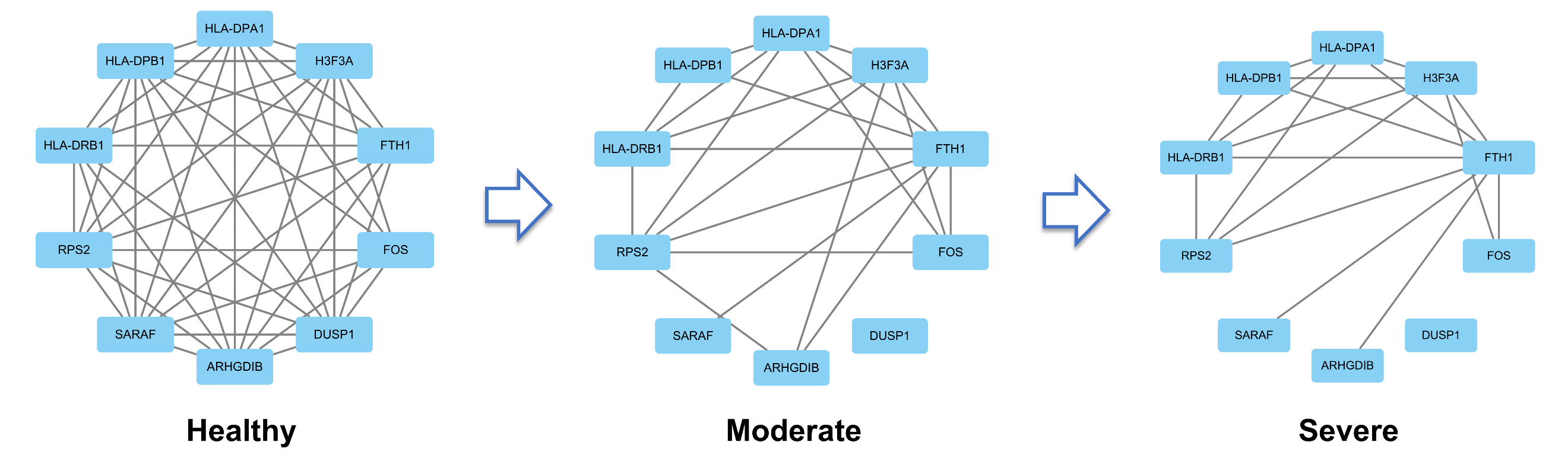


**Supplementary Figure S3.** CCSNs of B cells with the 10 DDGs in the GSE155673 dataset. The edge between two genes implies a direct gene dependency.


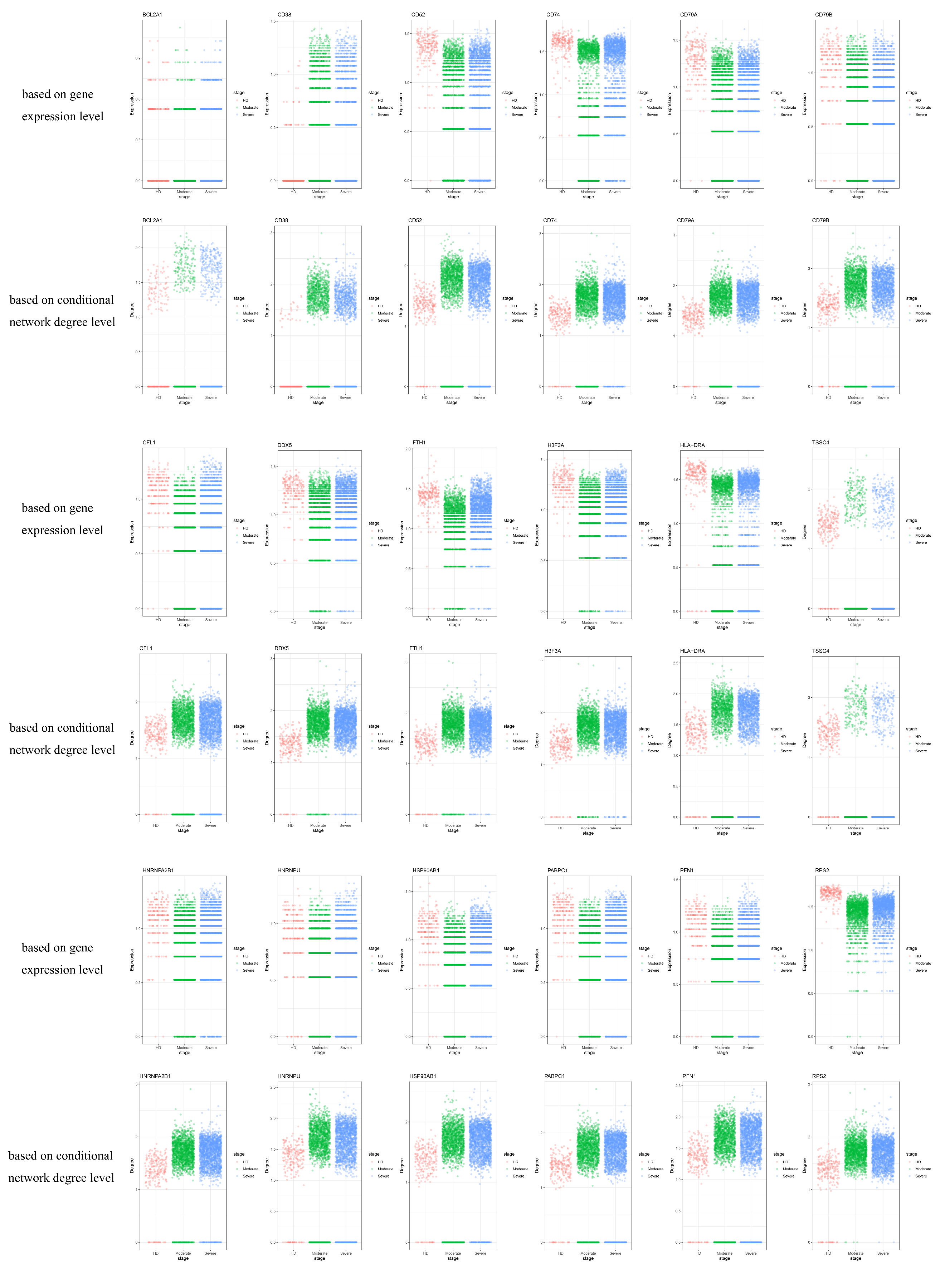


**Supplementary Figure S4.** The differences of gene expression level and conditional network degree level of some ‘dark’ genes in three different stages.

# Supplementary Note 5: The prognosis analysis of ‘dark’ genes


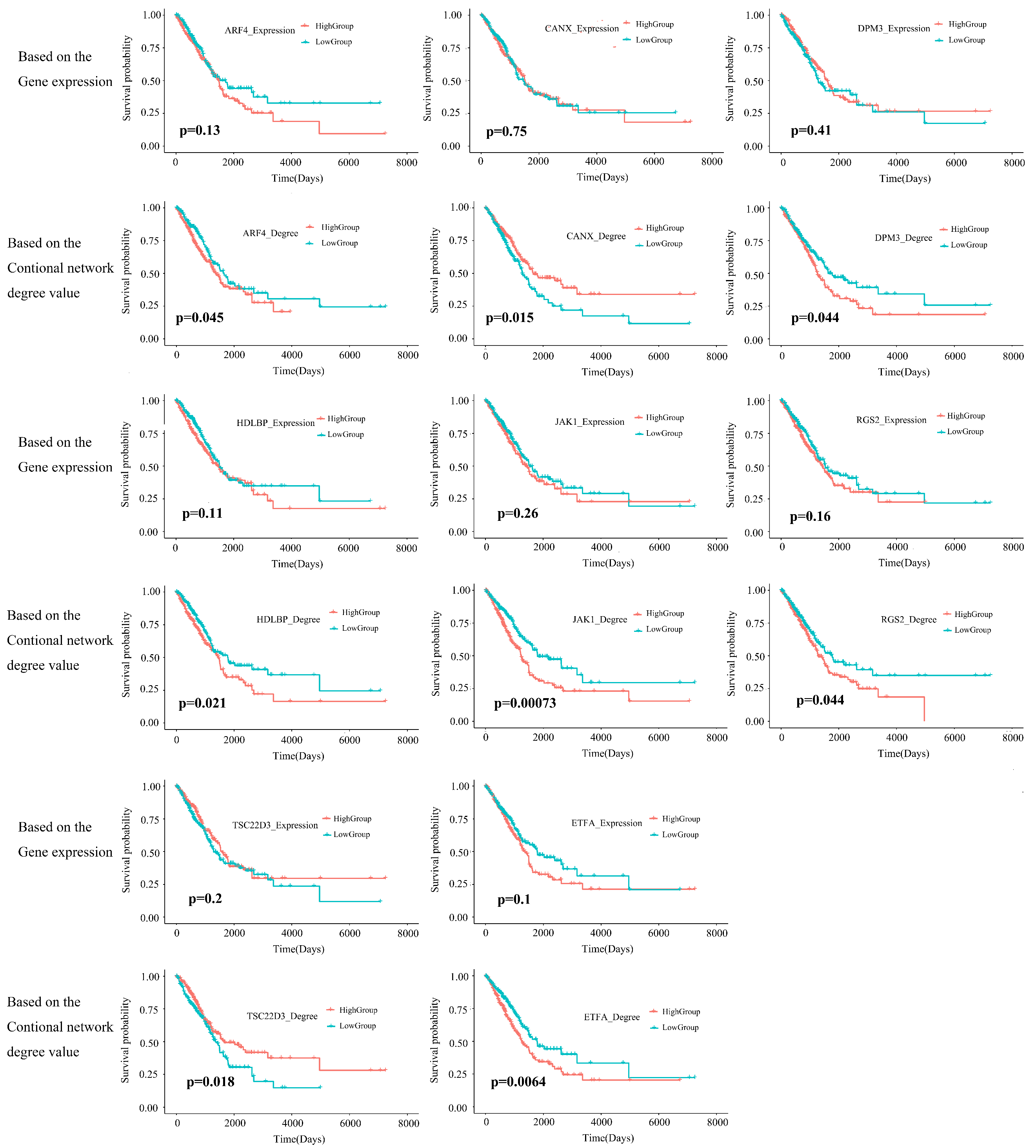


**Supplementary Figure S5.** The prognosis curve of ‘dark’ genes. According to Kaplan-Meier mapping, the prognostic significance of ‘dark’ genes in patients with LUAD was shown. High group represents patients with high gene expression or high conditional network degree values, and low group represents patients with low gene expression or low conditional network degree values. At the level of gene expression, the effect of gene expression on the survival rate of patients is very small, which does not reach statistical significance (P>0.05; log-rank test), but in the level of conditional network degree value of genes, the survival rate of patients is significantly different (P<0.05; log-rank test).

Due to the lack of corresponding prognostic data in the current research on COVID-19, the prognostic analysis of COVID-19 cannot be directly performed. However, through reviewing the literature, the high expression of some ‘dark’ genes is associated with the severity of COVID-19 patients. We believe that those genes whose high expression has bad effects on the disease have a poor prognosis. We found that the ‘dark’ genes ZBP1, JAK1, PDE4B, BRD2, TOP1, MYH9, VPS29, CD38 and IFP35 were related with poor prognosis. ZBP1 was expressed at higher levels in the immune cells of patients with poor prognosis during COVID-19 than those who fully recovered. Cell death was prevented in human cells in response to SARS-CoV-2 infection by knocking down the expression of ZBP1(4). JAK1 / JAK2 inhibitors, which prevented lung injury in severe covid-19, were suggested as a potential therapy against systemic inflammation in COVID-19(5). Treatment with an oral preferential inhibitor of the PDE4B subtype, prevented a decrease in lung function in patients with idiopathic pulmonary fibrosis (6). BRD2 inhibition could both block viral entry, through ACE2 downregulation, and acted as an ‘emergency brake’ for misregulated patient immune responses to COVID-19, via downregulation of ISGs (7). TOP1 inhibition had the potential for effective host-directed therapies against severe SARS-CoV-2 infection(8). MYH9 promoted authentic SARS-CoV-2 infection in human pulmonary cells (9). VPS29 gene facilitated coronavirus cell entry cells (10). SARS-CoV-2 exploited the upregulation of host ACE2R by increased expression of ISGs and CD38 in human lung epithelial cells, to enhance viral infection (11). SARS-CoV-2 infections resulted in elevated serum levels of IFP35. IFP35 was related to development of disease and might be used as a marker to predict prognosis (12). All these showed that the importance of ‘dark’ genes for the treatment and prognosis of COVID-19.

It has been reported that lung cancer patients are more susceptible to SARS-CoV-2 infection than normal individuals. Lung adenocarcinoma (LUAD) is one of the major histological subtypes of lung cancer and LUAD patients are excessively and aberrantly susceptible to SARS-CoV-2 infection (13). In order to explore the effect of ‘dark’ genes on COVID-19 patients, we also used LUAD data, which is closely related to COVID-19, to perform the prognosis of COVID-19 and reveal the effect of ‘dark’ genes on COVID-19 from another perspective important role.

We divided samples into high group and low group based on the median of gene expression or the median of conditional network degree values. By comparing the survival curves based on gene expression values and conditional network degree values (Fig. S5), we found that the survival curves based on conditional network degree values were significantly different. From the result of prognostic, ‘dark’ genes can be divided into two categories. Genes with higher conditional network degree values that have good prognosis are called ‘positive dark’ genes, such as TSC22D3 and CANX. While genes with higher conditional network degree values that have poor prognosis are called ‘negative dark’ genes, such as HCP5, JAK1, RGS2, ARF4, HDLBP, ETFA and DPM3. Therefore, the higher the conditional network degree value of ‘positive dark’ genes, the better the prognosis. The lower the conditional network degree value of ‘positive dark’ genes, the worse the prognosis. On the contrary, the higher the conditional network degree value of ‘negative dark’ genes, the worse the prognosis. The lower the conditional network degree value of ‘negative dark’ gene, the better the prognosis. The results of the ‘dark’ gene prognostic analysis based on LUAD indicate that some ‘dark’ genes are closely related to the development of LUAD. LUAD patients are also more susceptible to SARS-COV-2, and we reasoned that these ‘dark’ genes may be related to the development of COVID-19, which needs further investigation.

# Supplementary Note 6: Cell-cell communication analysis


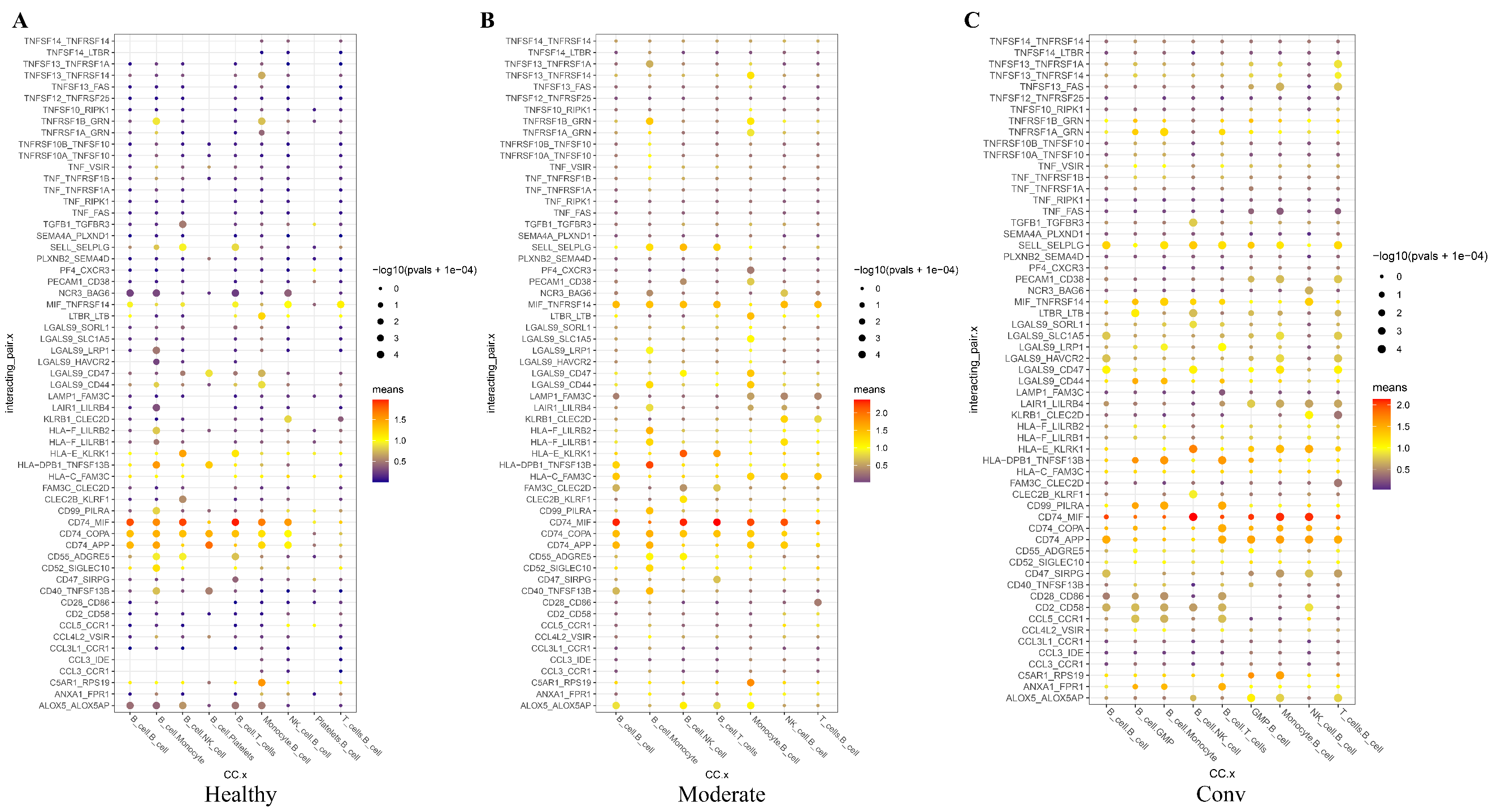


**Supplementary Figure S6.** **(A)**The B_cells-centric molecular interactions of peripheral immune cells in healthy-stage COVID-19 patient. P-values are represented by the size of the circle, and the ruler is shown on the right side of the graph. The color represents the average expression level of these two genes in the two cell clusters, and the darker the color, the higher the expression. The size of the bubble represents the -log10 value of the P value, and the larger the bubble, the more significant it is. **(B)** The B_cells-centric molecular interactions of peripheral immune cells in moderate-stage COVID-19 patient. **(C)** The B_cells-centric molecular interactions of peripheral immune cells in convalescent-stage COVID-19 patient.

The interaction of B cells with NK cells is most pronounced during normal, moderate, and convalescent stages. Natural killer (NK) cells play a critical role in modulating the immune response. Preliminary studies in COVID-19 patients with severe disease suggest a reduction in NK cell number and function, resulting in decreased clearance of infected and activated cells, and unchecked elevation of tissue-damaging inflammation markers (14). In the three stages, the most interacting pairing of B cells with other cell types is CD74_MIF (Fig. S6), which plays an important role in the process of inflammatory diseases.

# Supplementary Note 7: The underlying signaling mechanisms revealed by ‘dark’ genes

**

**

**Supplementary Figure S7.** Potential mechanisms revealed by the functional analysis of ‘dark’ genes, DDGs and DEGs. **(A)** Regulation of related ‘dark’ genes, DEGs and DDGs in the JAK-STAT signaling pathway. **(B)** Regulation of related ‘dark’ genes, DEGs and DDGs in the PI3K-AKT signaling pathway. **(C)** Regulation of related ‘dark’ genes, DEGs and DDGs in the MAPK signaling pathway.

**Some ‘dark’ genes and DDGs encode key proteins in the JAK-STAT pathway, two of which have antiviral effects, and another protein may have antiviral effects**

The 2’-5’OAS and PKR were the earliest discovered active protein kinases induced by double-stranded RNA (dsRNA), which relied on their respective functions to inhibit viral protein synthesis and viral infection (15). OAS induced by IFNs was inactive at first, and could only exert antiviral function after activation of dsRNA (16). After activation, OAS could induce ATP hydrolysis to generate 2’-5’ oligoadenylates, thereby activating ribonuclease L (RNase L). Activated RNase L could degrade virus and host RNA, inhibit protein synthesis, and play an antiviral function (17). PKR was able to be activated by dsRNA produced by the replication process after the virus invades the cell. The protein was originally in an inactive state in the cytoplasm, and when the double-stranded RNA of the virus was recognized, the protein phosphorylated and formed a dimer to activate the translation initiation factor eIF2α (Eukaryotic initiation factor 2 α, eIF2α) (18). Activation of this translation initiation factor not only inhibited the translation of viral mRNA to hinder the synthesis of viral elements, but also regulated the cell itself and the autophagy induced by the virus to antagonize the virus (19-21). PKR could upregulate IFNs by phosphorylation activating NF-kB, causing local tissues to rapidly stress viral invasion. In addition, PKR could cause apoptosis and thus hinder the spread of the virus (22).

Genetic studies of human populations had shown that a polymorphism in the MxA gene was correlated with increased susceptibility to HCV56 [37], HBV57 and measles virus. Mx proteins were expressed by various cell types in peripheral tissues, for example, by endothelial cells and immune cells, including peripheral blood mononuclear cells, plasmacytoid dendritic cells and myeloid cells [38]. Of the Mx family of proteins, only human MxA had been shown to have antiviral activity [23]. Mechanism of antiviral of MxA was following stimulation with type I interferons (IFNs), MxA expression was induced through an IFN-stimulated response element (ISRE) in the gene promoter. The MxA protein accumulated in the cytoplasm on intracellular membranes (such as the endoplasmic reticulum, ER) as oligomers formed by association between the leucine zipper (LZ) domain and central interactive domain of the protein. Following viral infection, MxA monomers were released and bind viral nucleocapsids or other viral components, to trap and then degrade them. This was a potent antiviral measure, which effectively prevents the generation of viral mutants that escape Mx-mediated antiviral mechanisms (23). Therefore, MxA might also be activated by SARS-CoV-2 to exert antiviral functions

**The protein p21 encoded by the ‘dark’ gene CDKN1A is a key regulator of the COVID-19 infection-related signaling pathway (JAK-STAT signaling pathway and PI3K-AKT signaling pathway). Elevated expression levels of proteins encoded by some DDGs are directly correlated with the severity of COVID-19 patients.**

In the JAK-STAT signaling pathway (Fig. S7A), the binding of cytokines to IL receptors (IL4R, IL6R, IL7R, IL10RA, and IL10RB) caused dimerization of the receptor molecules. However, these membrane receptors might not have kinase activity themselves, so they couldn’t directly activate downstream pathways, and they also needed to rely on the ‘dark’ gene JAK1 to encode kinase JAK. As the receptor dimerizes, the two JAK kinase molecules coupled to the receptor come close to each other and phosphorylate each other, and the intracellular terminus of the receptor was also phosphorylated. The phosphorylation of the receptor created a docking site for STAT and binds to IRF9, a member of the IRF (Interferon Regulatory Factor-9) family. Activated STATs (STAT1, STAT6) dimers translocated to the nucleus with IRF9, binding directly to DNA in the nucleus, activating the expression of the ‘dark’ gene CDKN1A-encoded protein p21, causing p21 to be upregulated, which inhibited the cell cycle (Fig. S7A).

IL4R、IL6R and IL7R respectively encoded the alpha chain of the interleukin-4 receptor, a subunit of the interleukin 6 (IL6) receptor complex, a receptor for interleukin 7 (IL7). IL10RA and IL10RB co-encodes the receptor of interleukin 10 (IL10). It had been reported that IL-4, IL-6, IL-7 and IL-10 levels are elevated in patients with COVID-19, could accelerate the inflammatory process and were directly related to disease severity (24). Numerous studies have described abnormal levels of cytokines and chemokines in the patients and found that the key point in SARS-CoV-2 infection could be the depletion of antiviral defenses related to innate immune response as well as an elevated production of inflammatory cytokines (25). ‘Dark’ gene JAK1 encoded a membrane protein that is a member of a class of protein-tyrosine kinases (PTK). The gene played a crucial role in affecting the expression of genes that mediate inflammation. JAK1 was a key component of the interleukin-6 (IL-6)/JAK1/STAT3 immune and inflammation response and a therapeutic target for alleviating cytokine storms. It encoded kinase phosphorylates STAT proteins (signal transducers and activators of transcription) and induces STAT dimerization. DDG IRF9 is a member of the IRF (Interferon Regulatory Factor-9) family of proteins encoded by the differential degree gene IRF9, has been demonstrated in previous studies to play a key role in inflammation (26), autoimmune diseases (27), cardiovascular diseases (28), cell proliferation and immune cell regulation (29). A recent study demonstrated that IRF9 among many other pro-inflammatory genes holds a high significance during immune related COVID-19 response (30). Interestingly, IRF9 fused with STAT2 transactivation domain alone can induce antiviral state (31). SARS-CoV-2 was evolved to interfere and tackle with IRF9 in multiple ways. Viruses could antagonize IRF9 via blocking its nuclear sequestration, blocking its binding with DNA and via promoting its degradation (32). The ‘dark’ gene CDKN1A encoded a potent cyclin-dependent kinase inhibitor p21. The upregulation of p21 could inhibit the cell cycle.

In the PI3K-AKT signaling pathway, the protein HSP90 encoded by the ‘dark’ gene HSP90AB1 and a novel AKT kinase co-activator, TCL1, activate serine /Threonine-specific protein kinase AKT, which promoted AKT-induced cell survival and proliferation (33). AKT phosphorylation inhibited CDK inhibitor p21 and cyclin inhibitor p27 to promote cell proliferation. Phosphoserine/phosphothreonine binding protein 14-3-3 inhibited the proapoptotic Bcl-2 family members BAD and FOXO proteins. AKT was a major mediator of cell survival, inhibiting pro-apoptotic proteins such as BAD through direct phosphorylation or inhibiting pro-apoptotic signals generated by transcription factors such as FOXO. Simultaneously AKT phosphorylated and activated the transcription factors CREB and IKK, affecting cell survival (Fig. S7B).

Not only does p21 played an important role in cell cycle arrest, apoptosis, and transcriptional regulation, but also it was critical for cellular processes of differentiation, senescence, and DNA repair. P21 activated serine/threonine kinases (PAKs), some of the pathogenic related kinases in PAKs, the abnormal activation of which was involved in inflammation, infections, including bacteria and SARS-CoV-2 [40,41]. The kinase PAK1 in PAK had been reported to be effective against SARS-CoV-2 therapy in preclinical trials [41,42]. Studies on the ‘dark’ gene CDKN1A had found that expression of the senescence markers CDKN1A was significantly increased in epithelial ciliated and club cells from patients with severe COVID-19 compared with those with moderate disease and with healthy control subjects [39]. All of these suggested that p21 played an important regulatory role in the transmission mechanism of COVID-19.

Essential for cell survival, the protein HSP90 encoded by the ‘dark’ gene HSP90AB1 was molecular chaperons required for conformational stabilization and trafficking of numerous client proteins. HSP90AB1 was also a potential biomarker for LUAD (34). Tang et al. found that LUAD (lung adenocarcinoma) patients were more susceptible to SARS-CoV-2 infection (35), so HSP90AB1 might be associated with COVID-19 infection and might become a potential biomarker for COVID-19. Functional HSP90 was required for the stability of AKT, a serine-threonine kinase phosphorylated in response to growth factor stimulation (36). AKT could directly phosphorylate a variety of transcription factors. By regulating these transcription factors, AKT could inhibit the expression of apoptotic genes and enhance the expression of anti-apoptotic genes, thereby promoting cell survival. AKT controlled a variety of downstream signaling pathways, and the downstream target proteins of AKT phosphorylate p21 would result in the down-regulation of p21. Down-regulation of p21 expression could stimulate the activity of CDKs, thereby promoting the progression of the cell cycle.

**The proteins encoded by the ‘dark’ genes TRADD, FOS and DDGs RELB, JUN, IKBKG are involved in the MAPK signaling pathway, which is a potential signaling pathway for the spread and development of COVID-19.**

There was an indirect role of mitogen-activated protein kinase (MAPK) in the Coronavirus disease-COVID-19[48-50]. We mined the MAPK signaling pathway and found that ‘dark’ genes were involved in the encoding of proteins in this pathway. The protein TRADD encoded by the ‘dark’ gene TRADD indirectly activated the protein CASP encoded by DDG CASP3, and played a central role in the execution stage of apoptosis. Tpl2/Cot encoded by DDG MAP3K8 reacted to stimulation by the TLR/IL-1 receptor superfamily and phosphorylation activates ERK, JNK and NFkB. The protein MKP encoded by the ‘dark’ gene DUSP1 could dephosphorylate the MAP kinase MAPK1/ERK2, which played a role in the negative regulation of cell proliferation. ERK phosphorylation activated STMN1 encoded by ‘dark’ gene STMN1. Continuous activation of ERK and RSK2 led to phosphorylation and transactivation of CREB. ERKs could also phosphorylate transcription factors such as c-fos within the nucleus, thereby participating in the regulation of cell proliferation and differentiation. Activated JNK activated its downstream transcription factors AP1 and JUND through phosphorylation, which were involved in cell proliferation, differentiation and inflammatory responses (Fig. S7C).

MAPK was a main cell signaling pathway that was known to be activated by a wide variety of viruses [43]. The activation of NFkB was a hallmark of many viral infections, and triggering of NFkB activation was particularly relevant during infection with viruses that had NFkB binding sites in their genome [47]. NFkB expression was upregulated, causing a variety of downstream proliferation-promoting genes and anti-apoptotic mechanisms as well as inflammatory responses. As with many other diseases, inflammation was a key feature of COVID-19. DUSP1 inhibited MAPK signaling and pro-inflammatory cytokine production [47]. The downregulation of the protein MKP encoded by DUSP1 increased the expression of ERK, thereby indirectly promoting the upregulation of CREB and c-fos expression, which caused cell proliferation and differentiation. The FOS proteins have been implicated as regulators of cell proliferation, differentiation, and transformation. In some cases, expression of the FOS gene had also been associated with apoptotic cell death. At the same time, FOS was also involved in the coronavirus infection [44]. The activator protein-1 (AP1) was a dimeric transcription factor formed by members of the JUN and FOS protein family [45]. The coronavirus N protein could activate AP1 signal transduction pathway was shown in [46]. In conclusion, the MAPK signaling pathway involved in ‘dark’ genes was a potential signaling pathway for the spread and development of SARS-COV-2.

# References

1. Li, L. et al. c-CSN: Single-cell RNA sequencing data analysis by conditional cell-specific network. Genomics, proteomics & bioinformatics. **19**, 319-329 (2021).
2. Cheng, W., Chen, G., Jia, H., He, X. and Jing, Z. DDX5 RNA Helicases: Emerging Roles in Viral Infection. Int. J. Mol. Sci. **19**, 1122(2018).
3. 3.Shahriari-Felordi, M., Alikhani, H.K., Hashemian, S.R., Hassan, M. and Vosough, M. Mini review ATF4 and GRP78 as novel molecular targets in ER-Stress modulation for critical COVID-19 patients. Mol. Biol. Rep. **49**, 1545-1549(2022).
4. Karki, R. et al. ZBP1-dependent inflammatory cell death, PANoptosis, and cytokine storm disrupt IFN therapeutic efficacy during coronavirus infection. Science Immunology. eabo6294 (2022).
5. Levy, G., Guglielmelli, P., Langmuir, P. & Constantinescu, S. JAK inhibitors and COVID-19. J. Immunother. Cancer. **10**, e002838 (2022).
6. Richeldi, L. et al. Trial of a Preferential Phosphodiesterase 4B Inhibitor for Idiopathic Pulmonary Fibrosis. N. Engl. J. Med. **386**, 2178-2187 (2022).
7. Samelson, A. J. et al. BRD2 inhibition blocks SARS-CoV-2 infection by reducing transcription of the host cell receptor ACE2. Nature Cell Biology. 24, 24-34 (2022).
8. Ho, J. S. Y. et al. TOP1 inhibition therapy protects against SARS-CoV-2-induced lethal inflammation. Cell. **184**, 2618-2632.e17 (2021).
9. Chen, J. et al. Nonmuscle myosin heavy chain IIA facilitates SARS-CoV-2 infection in human pulmonary cells. Proc. Natl. Acad. Sci. U. S. A. **118**, e21110111182021(2021).
10. Poston, D., Weisblum, Y., Hobbs, A. & Bieniasz P. D. VPS29 exerts opposing effects on endocytic viral entry. mBio. **13**, e0300221 (2022).
11. Horenstein, A. L., Faini, A. C. & Malavasi, F. CD38 in the age of COVID-19: a medical perspective. Physiological Reviews. **101**, 1457-1486 (2021).
12. Yu, Y. et al. IFP35 as a promising biomarker and therapeutic target for the syndromes induced by SARS-CoV-2 or influenza virus. Cell Reports. **37**, 110126 (2021).
13. Uddin, M. N. et al. Expression of SARS-COV-2 cell receptor gene ACE2 is associated with immunosuppression and metabolic reprogramming in lung adenocarcinoma based on bioinformatics analyses of gene expression profiles. Chem. Biol. Interact. **335**, 109370 (2021).
14. Van, E. C. et al. Natural killer cell dysfunction and its role in COVID-19[J]. International journal of molecular sciences. **21**, 6351(2020).
15. Bai, S. Y., Yang, Q. & Qiu, H. J. Antiviral mechanisms of interferon-stimulated genes. Acta Microbiologica Sinica. 58, 361-371 (2018).
16. Hovanessian, A. G. Interferon-induced and double-stranded RNA-activated enzymes:a specific protein kinase and 2-’5’-oligoadenylate synthetases. Journal of Interferon Research. **11**, 199–205 (1991).
17. Floyd-Smith, G., Slattery, E. & Lengyel, P. Interferon action: RNA cleavage pattern of a (2′-5′) oligoadenylate—Dependent endonuclease. Science. **212**, 1030-1032 (1981).
18. Sudhakar, A. et al. Phosphorylation of serine 51 in initiation factor 2α (eIF2α) promotes complex formation between eIF2α (P) and eIF2B and causes inhibition in the guanine nucleotide exchange activity of eIF2B. Biochemistry. **39**, 12929-12938 (2000).
19. Feng, G. S. et al. Identification of double-stranded RNA-binding domains in the interferon-induced double-stranded RNA-activated p68 kinase. Proceedings of the National Academy of Sciences. **89**, 5447-5451 (1992).
20. Amici, C. et al. Inhibition of viral protein translation by indomethacin in vesicular stomatitis virus infection: Role of e if 2α kinase pkr. Cellular Microbiology. **17**, 1391-1404 (2015).
21. Tallóczy, Z. et al. Regulation of starvation-and virus-induced autophagy by the eIF2α kinase signaling pathway. Proceedings of the National Academy of Sciences. **99**, 190-195 (2002).
22. Wang, Y. et al. Inhibition of PKR protects against H2O2-induced injury on neonatal cardiac myocytes by attenuating apoptosis and inflammation. Scientific Reports, **6**, 1-11 (2016).
23. Sadler, A, J. & Williams, B. R. G. Interferon-inducible antiviral effectors[J]. Nature reviews immunology. **8**, 559-568 (2008).
24. Costela-Ruiz, V. J. et al. SARS-CoV-2 infection: The role of cytokines in COVID-19 disease. Cytokine Growth Factor Rev. **54**, 62-75 (2020).
25. Blanco-Melo, D. et al. Imbalanced host response to SARS-CoV-2 drives development of COVID-19. Cell. **181**, 1036-1045. e9 (2020).
26. Rauch, I. et al. Noncanonical effects of IRF9 in intestinal inflammation: more than type I and type III interferons. Molecular and Cellular Biology. **35**, 2332-2343 (2015).
27. Smith, S. et al. MicroRNA-302d targets IRF9 to regulate the IFN-induced gene expression in SLE. Journal of Autoimmunity. **79**, 105-111 (2017).
28. Jiang, D. S. et al. Interferon regulatory factor 9 protects against cardiac hypertrophy by targeting myocardin. Hypertension. **63**, 119-127 (2014).
29. Huber, M. et al. IRF9 prevents CD8+ T cell exhaustion in an extrinsic manner during acute lymphocytic choriomeningitis virus infection. Journal of Virology. **91**, e01219-17 (2017).
30. Bluyssen, H. A. R. & Levy, D. E. Stat2 is a transcriptional activator that requires sequence-specific contacts provided by stat1 and p48 for stable interaction with DNA. Journal of Biological Chemistry. **272**, 4600-4605 (1997)
31. Kraus, T. A. et al. A hybrid IRF9-STAT2 protein recapitulates interferon-stimulated gene expression and antiviral response. Journal of Biological Chemistry. **278**, 13033-13038 (2003).
32. Mishra, R. & Banerjea, A. C. SARS-CoV-2 spike targets USP33-IRF9 axis via exosomal miR-148a to activate human microglia. Front. Immunol. **12**, 656700 (2021).
33. Laine, J. et al. The protooncogene TCL1 is an Akt kinase coactivator. Molecular Cell. **6**, 395-407 (2000).
34. Uddin, M. N. et al. Expression of SARS-COV-2 cell receptor gene ACE2 is associated with immunosuppression and metabolic reprogramming in lung adenocarcinoma based on bioinformatics analyses of gene expression profiles. Chem. Biol. Interact. **335**, 109370 (2021).
35. Tang, B. et al. The Landscape of Coronavirus Disease 2019 (COVID-19) and Integrated Analysis SARS-CoV-2 Receptors and Potential Inhibitors in Lung Adenocarcinoma Patients. Front. Cell. Dev. Biol. **8**, 577032 (2020).
36. Piredda, M. L. et al. PML/RARA inhibits expression of HSP 90 and its target AKT. Br. J. Haematol. **184**, 937-948 (2019).
37. Hijikata M, Ohta Y, Mishiro S. Identification of a Single Nucleotide Polymorphism in the MxA Gene Promoter (G/T at nt –88) Correlated with the Response of Hepatitis C Patients to Interferon. Intervirology (2000) 43:124–127. doi: 10.1159/000025035
38. Fernández M, Quiroga JA, Martín J, Herrero M, Pardo M, Horisberger MA, Carreño V. In Vivo and In Vitro Induction of MxA Protein in Peripheral Blood Mononuclear Cells from Patients Chronically Infected with Hepatitis C Virus. J INFECT DIS (1999) 180:262–267. doi: 10.1086/314859
39. Lipskaia L, Maisonnasse P, Fouillade C, Sencio V, Pascal Q, Flaman J-M, Born E, Londono-Vallejo A, Le Grand R, Bernard D, et al. Evidence That SARS-CoV-2 Induces Lung Cell Senescence: Potential Impact on COVID-19 Lung Disease. Am J Respir Cell Mol Biol (2022) 66:107–111. doi: 10.1165/rcmb.2021-0205LE
40. Chan PM, Manser E. “PAKs in Human Disease.,” Progress in Molecular Biology and Translational Science. Protein Phosphorylation in Health and Disease. Academic Press (2012). p. 171–187 doi: 10.1016/B978-0-12-396456-4.00011-0
41. Berretta AA, Silveira MAD, Cóndor Capcha JM, De Jong D. Propolis and its potential against SARS-CoV-2 infection mechanisms and COVID-19 disease: Running title: Propolis against SARS-CoV-2 infection and COVID-19. Biomedicine & Pharmacotherapy (2020) 131:110622. doi: 10.1016/j.biopha.2020.110622
42. Maruta H, He H. PAK1-blockers: Potential Therapeutics against COVID-19. Medicine in Drug Discovery (2020) 6:100039. doi: 10.1016/j.medidd.2020.100039
43. Kumar R, Khandelwal N, Thachamvally R, Tripathi BN, Barua S, Kashyap SK, Maherchandani S, Kumar N. Role of MAPK/MNK1 signaling in virus replication. Virus Research (2018) 253:48–61. doi: 10.1016/j.virusres.2018.05.028
44. Salgado-Albarrán M, Navarro-Delgado EI, Del Moral-Morales A, Alcaraz N, Baumbach J, González-Barrios R, Soto-Reyes E. Comparative transcriptome analysis reveals key epigenetic targets in SARS-CoV-2 infection. npj Syst Biol Appl (2021) 7:21. doi: 10.1038/s41540-021-00181-x
45. Langer S, Singer CF, Hudelist G, Dampier B, Kaserer K, Vinatzer U, Pehamberger H, Zielinski C, Kubista E, Schreibner M. Jun and Fos family protein expression in human breast cancer: Correlation of protein expression and clinicopathological parameters. EJGO (2006) 27:345–352.
46. He R, Leeson A, Andonov A, Li Y, Bastien N, Cao J, Osiowy C, Dobie F, Cutts T, Ballantine M, et al. Activation of AP-1 signal transduction pathway by SARS coronavirus nucleocapsid protein. Biochemical and Biophysical Research Communications (2003) 311:870–876. doi: 10.1016/j.bbrc.2003.10.075
47. Goel S, Saheb Sharif-Askari F, Saheb Sharif Askari N, Madkhana B, Alwaa AM, Mahboub B, Zakeri AM, Ratemi E, Hamoudi R, Hamid Q, et al. SARS-CoV-2 Switches ‘on’ MAPK and NFκB Signaling via the Reduction of Nuclear DUSP1 and DUSP5 Expression. Front Pharmacol (2021) 12:631879. doi: 10.3389/fphar.2021.631879
48. Kanehisa M, Goto S. KEGG: kyoto encyclopedia of genes and genomes[J]. Nucleic acids research, 2000, 28(1): 27-30.
49. Kanehisa M. Toward understanding the origin and evolution of cellular organisms[J]. Protein Science, 2019, 28(11): 1947-1951.
50. Kanehisa M, Furumichi M, Sato Y, et al. KEGG for taxonomy-based analysis of pathways and genomes[J]. Nucleic Acids Research, 2023, 51(D1): D587-D592.
