# Supplementary material for: Potential network markers and signaling pathways for B cells of COVID-19 based on single-cell condition-specific networks
Source: BMC Genomics. 2023 Oct 18;24:619. doi: 10.1186/s12864-023-09719-1 (PMC10583333; doi:10.1186/s12864-023-09719-1)
Supplement: Supplementary file 1 — Additional file 1: Table S1A. Differential expression genes obtained from healthy and moderate comparative analysis based on GEM. Table S1B. Differential expression genes obtained from healthy and severe comparative analysis based on GEM. Table S1C. Differential expression genes obtained from healthy and convalescent comparative analysis based on GEM. Table S1D. Differential degree genes obtained from healthy and moderate comparative analysis based on CNDM. Table S1E. Differential degree genes obtained from healthy and severe comparative analysis based on CNDM. Table S1F. Differential degree genes obtained from healthy and convalescent comparative analysis based on CNDM. Table S1G. DGs. Table S1H. 'dark' genes. Table S1I. DDGs.Table S1J. DEGs. Supplementary Note 1. Construct of CCSN and obtain CNDM from CCSN. Supplementary Note 2. Network-based cell clustering, gene dimension-reduction analysis and cell counts. Supplementary Note 3. ‘Dark’ genes revealed by CNDM. Supplementary Note 4. Validation of experimental results based on the COVID-19 single-cell sequencing dataset GSE155673. Supplementary Note 5. The prognosis analysis of ‘dark’ genes. Supplementary Note 6. Cell-cell communication analysis. Supplementary Note 7. The underlying signaling mechanisms revealed by ‘dark’ genes. [file 12864_2023_9719_MOESM1_ESM.zip › Revised supplementary information/Supplementary Table/Supplementary Table S5.docx]

**Table S5.** **The comparation between the study and other study**

|  | **Study data** | **The type of data being analyzed** | **Basic analytical method** | **Findings** | **Comparison** |
| --- | --- | --- | --- | --- | --- |
| Li et al. (this work) | The raw scRNA-seq data of PBMCs from 13 patients and 5 healthy controls were downloaded from the GSA (accession HRA000150). The 13 patients with COVID-19 were classified into three clinical conditions: moderate (n = 7), severe (n = 4) and convalescent (conv; n = 6, of whom 4 were paired with moderate cases). | GEM; CNDM | CCSN; Cell clustering; Multiple dataset integration; Cell-type annotation and cluster marker identification; DEG identification and functional enrichment; DDGs identification and functional enrichment; | 1. FTH1 might be a potential network marker for COVID-19.  2. MIF and CD74 were emerging as attractive candidates for immunotherapy force targets.  3. Based on the CNDM of B cells, the hub genes and ‘dark’ genes closely related to COVID-19 were revealed.  4. The proteins encoded by ‘dark’ genes complement some missing links in COVID-19 and these signaling pathways played an important role in the growth and activation of B cells. | 1. Our research has not only analyzed based on the traditional GEM, but also based on the CNDM.  2.Our study can obtain not only DEGs, but also DDGs and even ‘dark’ genes.  3.Our algorithm first constructs CCSN for each cell and then transforms CNDM. This would be more robust and alleviate dropout events to some extent.  4.Not only did we perform traditional analyses such as clustering, identification of differential genes, and enrichment analysis, but we also conducted specialized analyses including prognosis analysis, protein validation, and pathway analysis. We can get more conclusions from the analysis. |
| Zhang et al. (2020) | The raw scRNA-seq data of PBMCs from the GSA (accession HRA000150). | GEM | Cell clustering; Multiple dataset integration; Cell subclustering; Cell-type annotation and cluster marker identification; DEG identification and functional enrichment; Defining cell state scores; TCR and BCR V(D)J sequencing and analysis; Plasma cytokine detection. | 1. Most cell types in patients with COVID-19 showed a strong interferon-α response and an overall acute inflammatory response.  2. Intensive expansion of highly cytotoxic effector T cell subsets was associated with convalescence in moderate patients.  3. In severe patients, the immune landscape featured a deranged interferon response, profound immune exhaustion with skewed T cell receptor repertoire and broad T cell expansion. | 1.This research only analyzed based on the traditional GEM.  2.Only the DEGs could be obtained.  3.It simply defines the cell state score.  4. This study only conducted traditional analyses and did not perform further downstream analysis to discuss the significant role of proteins encoded by differentially expressed genes in COVID-19 |
| Zhang et al. (2021) | The raw scRNA-seq data of PBMCs from the GSA (accession HRA000150). | GEM | Cell clustering; Multiple dataset integration; Cell subclustering; Cell-type annotation and cluster marker identification; DEG identification and functional enrichment; Construct the gene co-expression network of the cells. | EEF1A1 is expected to serve as a diagnosis and treatment marker of SARS-COV-2 infection. | 1. This research only analyzed based on the traditional GEM.  2.Only the DEGs could be obtained.  3.Only that the gene co-expression network was constructed.  4.This study only conducted traditional analyses and did not perform further downstream analysis to discuss the significant role of proteins encoded by differentially expressed genes in COVID-19. |
| Shi et al. (2021) | The raw scRNA-seq data of PBMCs from the GSA (accession HRA000150). | GEM | Cell clustering; Multiple dataset integration; Cell subclustering; Cell-type annotation and cluster marker identification; DEG identification and functional enrichment; Defining cell state scores; TCR analysis; Flow Cytometry. | 1. DEGs of MAIT cells were involved in myeloid leukocyte activation and lymphocyte activation in patients with COVID-19.  2. MAIT cells are likely to be involved in the host immune response against SARS-CoV-2 infection. | 1.This research only analyzed based on the traditional GEM.  2.Only the DEGs could be obtained.  3.It simply defines the cell state score.  4.This study only conducted traditional analyses and did not perform further downstream analysis to discuss the significant role of proteins encoded by differentially expressed genes in COVID-19. |

**Note:**

GEM: Gene expression matrix

CNDM: conditional network degree matrix

DEG: differentially expressed genes

DDG: differential degree genes
